# RhoC Interacts with Integrin α5β1 and Enhances Its Trafficking in Migrating Pancreatic Carcinoma Cells

**DOI:** 10.1371/journal.pone.0081575

**Published:** 2013-12-03

**Authors:** Ningfeng Fiona Li, Emilios Gemenetzidis, Francis J. Marshall, Derek Davies, Yongwei Yu, Kristopher Frese, Fieke E. M. Froeling, Adam K. Woolf, Roger M. Feakins, Yoshiki Naito, Christine Iacobuzio-Donahue, David A. Tuveson, Ian R. Hart, Hemant M. Kocher

**Affiliations:** 1 Barts Cancer Institute - a CR-United Kingdom Centre of Excellence, Queen Mary University of London, Centre for Tumour Biology, London, United Kingdom; 2 Cancer Research United Kingdom London Research Institute, London, United Kingdom; 3 Changhai Hospital of Shanghai Second Military Medical University, Pathology Department, Shanghai, China; 4 Cancer Research United Kingdom Cambridge Research Institute, Cambridge, United Kingdom; 5 Barts and the London HPB Centre, The Royal London Hospital, London, United Kingdom; 6 Department of Pathology, The Johns Hopkins University School of Medicine, Baltimore, Maryland, United States of America; Thomas Jefferson University, United States of America

## Abstract

Human pancreatic ductal adenocarcinoma (PDAC) is characterized by early systemic dissemination. Although RhoC has been implicated in cancer cell migration, the relevant underlying molecular mechanisms remain unknown. RhoC has been implicated in the enhancement of cancer cell migration and invasion, with actions which are distinct from RhoA (84% homology), and are possibly attributed to the divergent C-terminus domain. Here, we confirm that RhoC significantly enhances the migratory and invasive properties of pancreatic carcinoma cells. In addition, we show that RhoC over-expression decreases cancer cell adhesion and, in turn, accelerates cellular body movement and focal adhesion turnover, especially, on fibronectin-coated surfaces. Whilst RhoC over-expression did not alter integrin expression patterns, we show that it enhanced integrin α5β1 internalization and re-cycling (trafficking), an effect that was dependent specifically on the C-terminus (180-193 amino acids) of RhoC protein. We also report that RhoC and integrin α5β1 co-localize within the peri-nuclear region of pancreatic tumor cells, and by masking the CAAX motif at the C-terminal of RhoC protein, we were able to abolish this interaction *in vitro* and *in vivo*. Co-localization of integrin α5β1 and RhoC was demonstrable in invading cancer cells in 3D-organotypic cultures, and further mimicked *in vivo* analyses of, spontaneous human, (two distinct sources: operated patients and rapid autopsy programme) and transgenic murine (LSL-KrasG12D/+;LSL-Trp53R172H/+;Pdx-1-Cre), pancreatic cancers. In both cases, co-localization of integrin α5β1 and RhoC correlated with poor differentiation status and metastatic potential. We propose that RhoC facilitates tumor cell invasion and promotes subsequent metastasis, in part, by enhancing integrin α5β1 trafficking. Thus, RhoC may serve as a biomarker and a therapeutic target.

## Introduction

The RhoA-like sub-family molecules of small GTPases (RhoA, RhoB and RhoC) share nearly 84% amino acid sequence homology, differing predominantly in their C-terminus domain [1]. Until recently, this sequence homology has prevented specific causality being attributed to each Rho GTPase for their explicit roles in distinct biological functions [2–4]. Increased expression of RhoC has been implicated in the metastatic process in pathologically distinct human cancers [5–7]. *In vivo* functional investigations indicated that RhoC, although dispensable for embryonic/postnatal development and tumor initiation, was critical for tumor metastasis [8,9]. Recent *in vitro* analyses have suggested that RhoC may mediate cancer cell invasion via control of other molecules, such as formin (FMNL2 [10] and FMNL3 [11]) at lamellipodia or through spatial resolution of RhoC at invadopodia [12] or, possibly, via upstream regulators such as Notch-1 [6], mir10b [3], p38γ-mediated RhoC ubiquitination [13] or RhoGDP dissociation inhibitor α (RhoGDIα) [14]. However, the relevant molecular mechanisms distinct from modulation of GTPase-like activity, specifically including cues from the micro-environment, which drive the RhoC-induced cellular phenotypic changes and metastatic proclivity, remain largely unknown. 

The subcellular localization of RhoC (mainly in the cytosol but particularly associated with the submembranous actin network, endoplasmic reticulum and additional compartments) has suggested a potential role in secretory granule exocytosis [15]. Similar to RhoA, RhoC undergoes post-translational modification of the C-terminus by CAAX motif dependent geranylgeranylation, with subsequent carboxymethylation, leading to the generation of an hydrophobic end facilitating its membrane localization [1]. Tagging or deleting the C-terminus of RhoC may disrupt such modifications, resulting in inefficient membrane localization. In this study, we over-expressed wild-type full-length human RhoC cDNA (nRhoC), as well as its C-terminus-deleted (nDCT) or tagged (cRhoC) forms, in human pancreatic ductal adenocarcinoma (PDAC) cell lines to investigate the intracellular localization and downstream mechanisms during cell migration and invasion. We demonstrate, in this report, a direct involvement of RhoC with trafficking and signaling of integrin α5β1 in invading pancreatic cancer cells. 

## Materials and Methods

### Cell culture and reagents

Capan1, Panc0403, HPAF and other pancreatic cancer cell lines were obtained (ATCC, LGC Standards, Middlesex, UK), STR profiled (Table S1, LGC Standards) and maintained as described previously [16]. Transfected cell lines were cultured in complete growth medium with 10µg/ml of Blasticidin (for Capan1; Invitrogen, Paisley, UK) or 150μg/ml of Hygromycin (for HPAF, Panc0403; Gibco, Paisley, UK). 10µg/milliliter fibronectin (Sigma-Aldrich, Dorset, UK) was used for coating. 

### Antibodies

Table S2.

### Plasmids

Human full length RhoC cDNA (Cat. No. TC127513) was from OriGene technologies (Rockville, MD, USA); In-Cell Labelling reagents from Invitrogen (Paisley, UK). Primers for PCR generation of RhoC constructs are listed (Table S3). Full length RhoC PCR product and C-terminal 180-193 amino acids deleted product were cloned into Mammalian Lumio^TM^ Gateway vectors (Invitrogen, Paisley, UK), with Lumio and V5 tag at the N-terminus to generate nRhoC and nDCT plasmids; and, with the tags at the C-terminus, to generate the cRhoC plasmid (Figure S1). RhoC was also cloned into vector plasmid pSecTag2B as an additional control.

We generated stable RhoC over-expressing cell lines, nRhoC, cRhoC and nDCT by lipofectamine transfection of these cDNAs respectively (nEV: control vector cell line, without RhoC cDNA but with sequences of a Chloramphenicol resistance gene and *ccdB* gene) into the Capan1 cells and expression was confirmed at both mRNA and protein levels, Figures S1F, S2A-C). Mass cultures, as well as single clones, of the transfected cells were maintained for later experiments.

Two small hairpin RNA interference (shRNA) sequences (Table S4) were generated targeting sequences of RhoC and introduced into pSilencer-hygro vector (Ambion, Warrington, UK) to transfect into the Panc0403, HPAF cells. Mass culture of the population with lower RhoC expression levels was maintained (Figure S2D-F).

### Cell spreading and Laser Scanning Cytometry (LSC)

Cells were plated on fibronectin coated coverslips and allowed to spread for 60 minutes before fixation in 4% formaldehyde. F-actin was stained with phalloidin and DNA with TO-PRO-3 iodide (2 μM; Invitrogen, Paisley, UK). A Laser Scanning Cytometer equipped with an Olympus BX50 microscope at 20X magnification was used for image capture. After elimination of cell clumps, the spread area of at least 1000 cells was calculated using Wincyte software (CompuCyte, Westwood, MA, USA). 

**Focal adhesion disassembly assay** [17], integrin **internalization and recycling assay** [18], **immunoprecipitation assays** and **immunofluorescence** [16] as well as **Organotypic culture assays** [19–21] were performed using well-validated methods described elsewhere (and Methods S1).

**Human PDAC tissue microarray** was constructed (ethical approval City and East London Local Research Ethics Committee 07/H0707/87) as described before [16]. The second tissue microarray was obtained from the Johns Hopkins rapid autopsy programme in an ethically approved manner [22].

**Transgenic mouse model tissue samples** from PDAC KPC (LSL-KrasG12D/+;LSL-Trp53R172H/+;Pdx-1-Cre [23]) mice (n=8) were used. All transgenic mice, as described, were generated at the Cambridge Research Institute under Home Office (UK) and Cancer Research UK Cambridge Research Institute guidelines.

### Statistics

All *in vitro* experiments were repeated on at least three independent occasions in triplicate for each condition. Normally (paired or unpaired Student’s-t test, ANOVA) or non-normally (Mann-Whitney test or Wilcoxon signed-rank test) distributed data were analyzed using SPSS 14.0 software (Surrey, UK) or GraphPad Prism5 software (La Jolla, CA, USA). 

## Results & Discussion

### RhoC over-expression decreased cell adhesion and promoted cell migration

**Figure 1:**
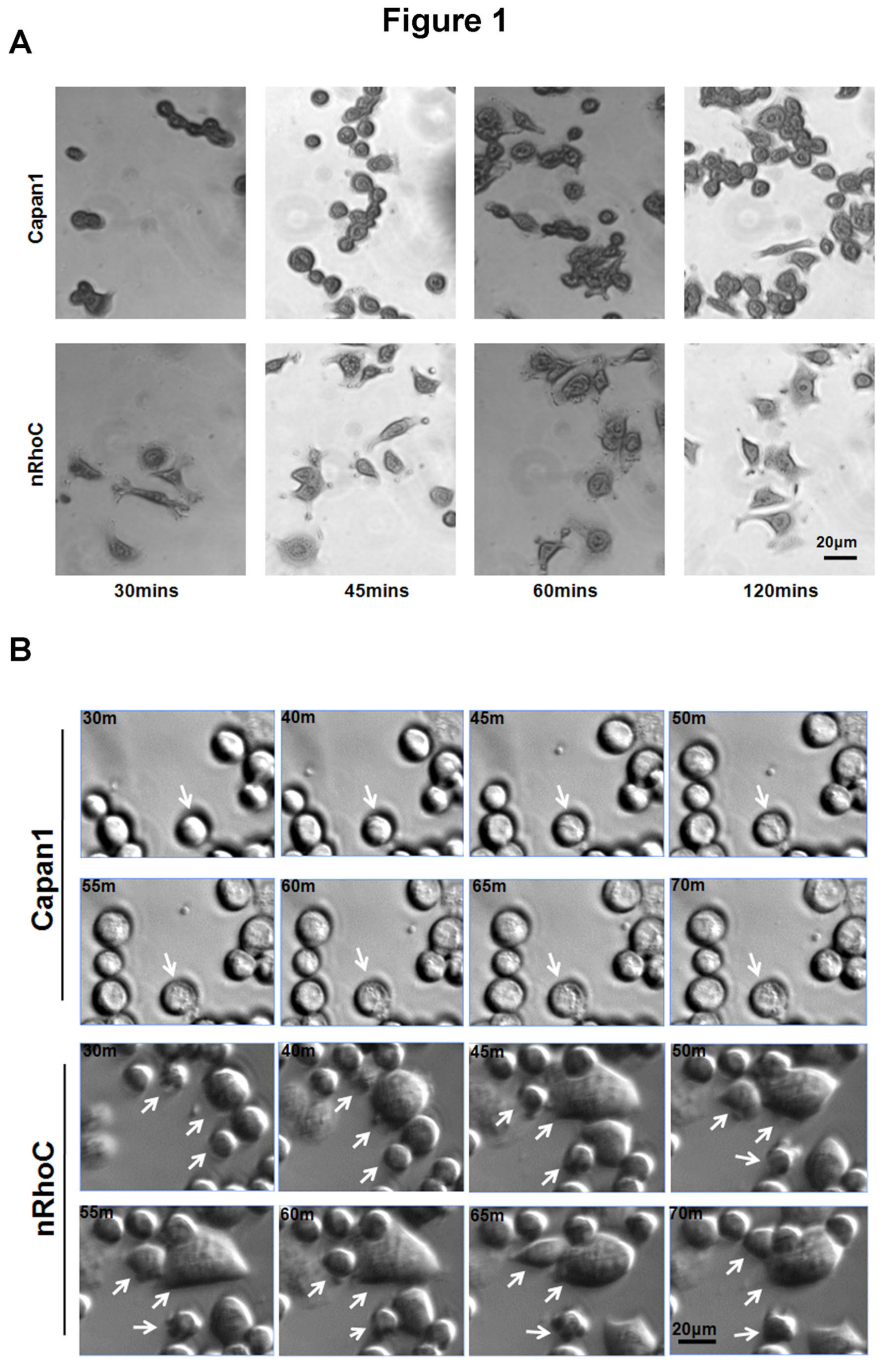
RhoC overexpression causes rapid spreading and movement of Capan1 cells. Initial screen of PDAC cell lines revealed low expression levels of RhoC protein in Capan1 pancreatic cancer cells which were then transfected with RhoC constructs (nRhoC, Figures S1, S2). (A) Cells were plated on the fibronectin-coated surface and incubated at 37°C till the indicated time-point, then fixed and stained with Crystal Violet before imaging with Zeiss Axiovert 200M microscope. nRhoC cells (Capan1 cells with N-terminus tagged RhoC overexpression) spread more within the first two hours as compared to the rounded parental Capan1 cells. (B) A selection of images from time-lapse videos (See videos S1, S2, and S3 for detailed information) of cell movement during two hours after plating on fibronectin-coated surface. These images demonstrate that nRhoC transfected cells had accelerated cell spreading and contraction, associated with rapid movement, as compared to Capan1 cells (arrows). Time of capture is indicated at the top-left corner of each image (m= minutes). Scale bar: 20µm.

**Figure 2:**
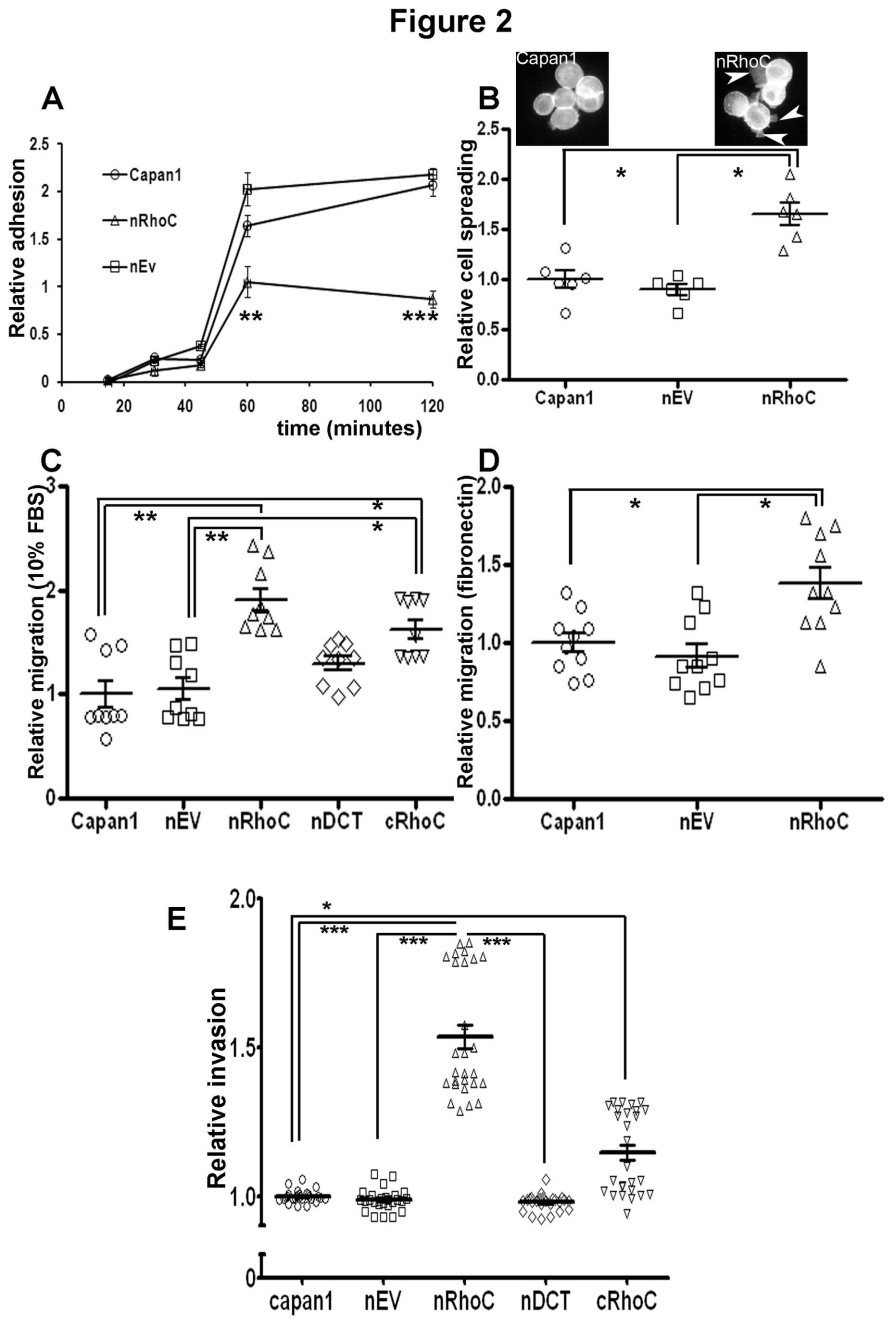
RhoC over-expression altered cellular adhesion, spreading and motility. (A) nRhoC cells (Capan1 cells with N-terminus tagged RhoC overexpression) showed decreased adhesion to fibronectin as compared to parental Capan1 cells or control plasmid / empty vector transfected cells (nEV) after one hour which was sustained up to two hours. (B) nRhoC cells also demonstrated a significant increase of cell spreading at an hour of plating compared with parental Capan1 and nEV cell lines. Insets show representative spreading morphology of nRhoC (arrowheads) as compared with Capan1 cells. (C) nRhoC cells showed a significant increase of cell migration across Transwell inserts towards 10% FBS as compared with parental Capan1 or nEV cell line. In addition, cRhoC (C-terminal tagged: CAAX-motif masked) cell line demonstrated enhanced migration, while the nDCT (C-terminus deleted) cell line did not show any difference from the two control (Capan 1, nEV) lines. (D) nRhoC cells showed a significant increase of cell migration across Transwell inserts through Fibronectin as compared with parental Capan1 or nEV cell line. (E) nRhoC cells demonstrated a significant increase of invasion across a Matrigel plug at 48 hours compared to parental Capan1, nDCT and nEV cell lines. cRhoC cells demonstrate a marginal increase in invasion as compared to parental Capan1 cells only. ** p<0.001, * p<0.01, Student’s t-test or ANOVA as applicable. Individual data points represent technical/biological repeats with summary statistics represented by mean ± SEM.

Relatively low (Capan1) and high (Panc0403, HPAF) expressors of endogenous RhoC levels, in comparison with hTERT immortalized normal human pancreatic ductal epithelial cells (DEC-hTERT [24]), were chosen from an initial screen for RhoA & RhoC protein levels in various PDAC cell lines [16] (Figure S1A, B). Capan1 cells were transfected with various constructs of RhoC (nRhoC, wild-type full length; cRhoC, CAAX motif masked RhoC; nDCT, C-terminal deleted RhoC; nEV, empty vector control) tagged with V5 and Lumio (Figures S1C-F, S2A-C). nRhoC cells displayed increased cell dissemination from individual colonies, adopting a looser growth pattern, compared with the parental Capan1 and nEV cells. Furthermore, time-lapse microscopy revealed an accelerated cell spreading, followed by immediate cell contraction and movement of nRhoC cells (Figure 1, Videos S1, S2, and S3). nRhoC cells also showed a significant reduction of cell adhesion on fibronectin-coated surfaces over the first hour after plating, compared with parental Capan1 and nEV control cells (Figure 2A). Although less adherent, most of the nRhoC cells spread more on fibronectin (Figure 2B) but not Collagen I (data not shown). These findings are in agreement with recent data suggesting a role for RhoC promoting cell movement and migration, and restricting lamellipodial broadening [11].

**Figure 3:**
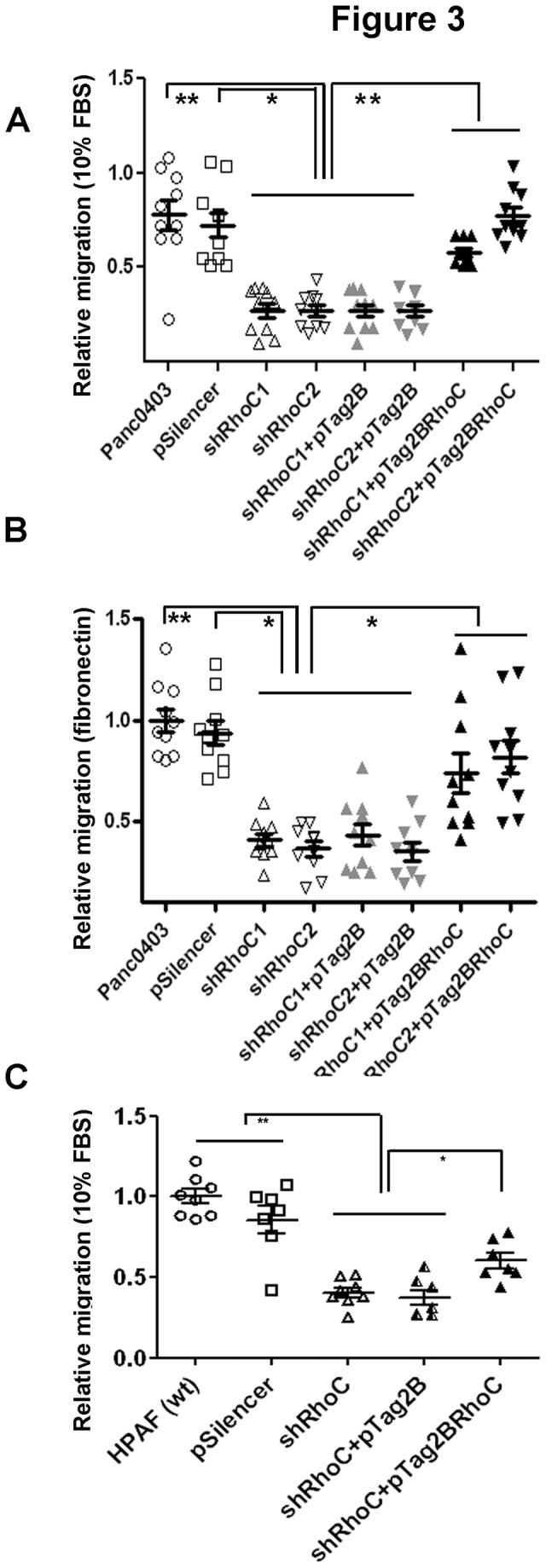
RhoC knockdown decrease cellular migration and is rescued by ectopic RhoC overexpression. Initial screen of PDAC cell lines revealed high expression levels of RhoC protein in HPAF and Panc0403 pancreatic cancer cells. Endogenous RhoC was silenced using shRNA (Figures S1, S2). shRhoC (stable RhoC knockdown: two different constructs were used: shRhoC1 and shRhoC2) resulted in a significant reduction of cell migration compared with the parental or pSilencer (empty vector control) cell lines towards 10% FBS (A) or through Fibronectin (B). Transient over-expression of RhoC in shRhoC cells (pTag2BRhoC) restored cell migration significantly and comparable to parental cell line. pTag2B is the empty vector control cell line. Similarly, another cell line (HPAF) demonstrated RhoC-dependent migration (C). Here the summary data of both ShRhoC are presented for migration towards 10% FBS. ** p<0.001, * p<0.01, ANOVA. Individual data points represent technical/biological repeats with summary statistics represented by mean ± SEM.

Concordant with these observations, nRhoC and cRhoC cells (as compared with Capan1, nDCT and nEV) cells exhibited significantly enhanced migratory (3D (Transwell) migration assay, both towards 10% FBS as well as through fibronectin) and invasive (through Matrigel plug) capacities (Figure 2C-E). When endogenous RhoC expression was down-regulated, by shRNA, in high expressors Panc0403 and HPAF (Figure S2D-F), we were able to diminish the migratory capacity of the cells, while re-introduction of wild-type RhoC partially restored this phenotype (Figure 3). Taken together, these data demonstrate that the levels of endogenous RhoC protein can significantly influence the capacity of pancreatic cancer cells to migrate, which is in agreement with the reported role of RhoC in promoting migration in different types of cancers [25,26]. Consistently, we observed that RhoC protein levels were dramatically elevated in cells which had migrated through the Transwell insert, regardless of whether RhoC was ectopically expressed (nRhoC cells, Figure S3), or its endogenous expression was knocked-down by shRNA (Panc0403 cells, Figure S4), suggesting that elevated RhoC protein levels enhanced cell migration.

### RhoC Over-Expression Accelerated Focal Adhesion Assembly Kinetics

Paxillin, a marker for focal adhesions was studied to define alterations of adhesion structures and/or their kinetics. No change in total paxillin expression, between Capan1, nEV and nRhoC cells, was detected. In three-dimensional reconstruction analyses, on stably adherent individual cells, there were no differences in paxillin distribution (potentially relatively stable adhesion complexes were assessed 10 hours after plating on non-coated surfaces by microscopy as well as Western blot: data not shown). 

**Figure 4:**
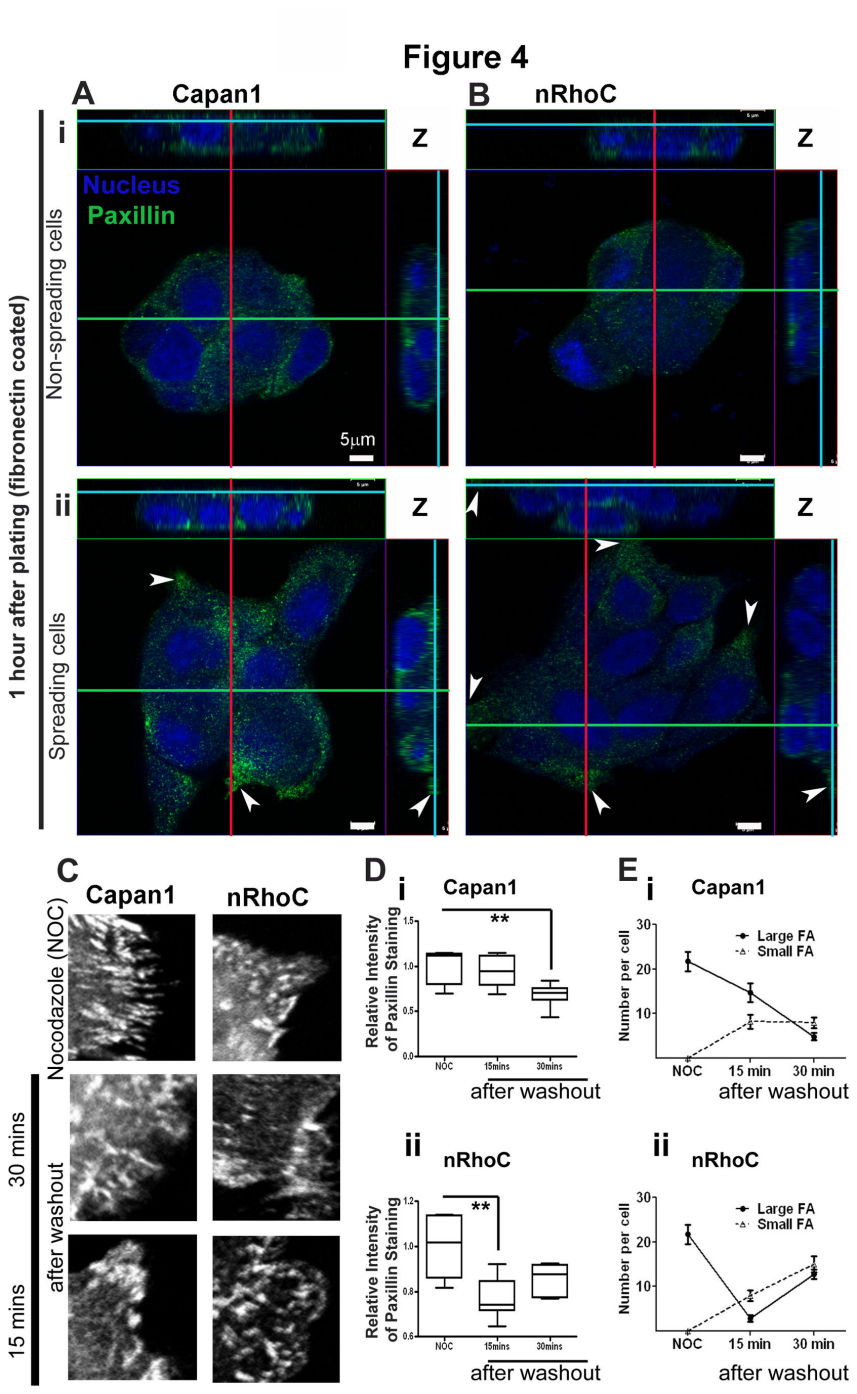
RhoC over-expression altered paxillin distribution and accelerated focal adhesion turnover. (A-B) Z-stacked confocal images showed differential distribution of paxillin staining (green) in ‘non-spreading’ (i) and ‘spreading’ (ii), Capan1 (A) and nRhoC (B) cells, 60 minutes after plating on the fibronectin-coated surface. Main images presented are in the XY plane with Z axis at the basal (ventral) aspects of cells as shown in the XZ and YZ sections at the top and right of composite images respectively (the cross-section of images in X, Y and Z axes are denoted by green, red and blue lines respectively). The obvious focal staining at the cell protrusion end of ‘spread’ Capan1 cells (Aii) was not present in the ‘spread’ nRhoC cells (Bii, arrowheads: cell protrusion ends). In the few ‘non-spreading’ cells, there were greater amounts of paxillin staining along the basal membrane of Capan1 cells (Ai) than that of nRhoC (Aii) cells. Scale bar: 5μm. (C-E) Focal adhesion ‘disassembly assay’ (NOC: nocodazole) showed that, compared to Capan 1, nRhoC cells had accelerated disassembly and re-assembly of mature focal adhesions (FA: large 4-5 μm) and small focal adhesion (1μm). Mean Intensity of peripheral paxillin staining showed similar levels 30 minutes after washout of the nocodazole treatment in Capan1 and nRhoC cells (Methods S1). However, while nRhoC cells had decreased the paxillin intensity levels significantly by 15 minutes after recovery from nocodazole, Capan1 cells took 30 minutes to achieve similar reduction, suggesting a quicker turnover of new focal adhesions in nRhoC cells. The size of focal adhesions after nocodazole treatment also demonstrates a quicker recovery/ re-assembly of stable large focal adhesions in nRhoC cells as compared to Capan1 cells. This could well explain the rapid movement and the increased cell spreading and decreased adhesions (Figures 1, 2, Videos S1, S2, and S3), as a result of introduction of RhoC in nRhoC cells. *p<0.05, Mann-Whitney test. Summary statistics in the box-whisker plot represented median ± inter-quartile range.

In contrast, at the early time-point (one hour), after plating on fibronectin-coated surfaces, when differences in adhesion/spreading occur as a result of RhoC over-expression (Figure 1,2), we found distinct differential distribution of paxillin staining in ‘spreading’ and ‘non-spreading’ nRhoC cells, compared with Capan1 parental cells. The individual ‘spreading’ Capan1 (Figure 4A,B) cells showed more paxillin staining localized to the cell periphery (protrusions) compared to nRhoC cells.

This differential distribution of paxillin as a result of exogenous RhoC expression suggested possible alterations, on fibronectin-coated surfaces, in focal adhesions turnover [27]. Using an established, well-validated semi-quantitative focal adhesion disassembly assay [17,28], we observed that after Nocodazole wash out, within 15 minutes, most of the nRhoC cells showed a reduction in both paxillin staining and focal adhesion size (disassembly of the over-sized (~4.5 μm length, [27])) and assembly of normal-sized (~1μm length) focal adhesions). In comparison, Capan1 cells did not demonstrate a similar reduction in focal adhesion staining until a later time (30 minutes, Figure 4C-E). This may suggest an altered kinetics of focal adhesions resulted from the ectopic expression of RhoC. The accelerated turn-over of focal adhesions in nRhoC cells may explain, at least in part, the decreased adhesion and increased spreading of these RhoC over-expressing cells (Figure 1,2).

### C-terminus of RhoC interacts with integrin α5β1

A major component of focal adhesion structures, especially in the presence of fibronectin, is constituted by members of the integrin family (heterodimers between α and β subunits [29]), such as integrin α5β1[30]. Recent studies have revealed that endo-exocytic trafficking of integrins, and especially that of integrins α5β1 and αvβ3, dictate the mode of migration of normal and carcinoma cells via activation of small GTPases and ROCK [31,32]. 

Accordingly, we sought to determine if the accelerated turnover of focal adhesions in nRhoC cells was due to the alteration of integrin expression and/or their trafficking upon stimulation with distinct extra-cellular matrix (ECM) proteins. Changes in morphology of nRhoC cells upon seeding on other ECM proteins, such as collagen and laminin, were not obvious (data not shown, (16)); hence, in this study, we did not investigate the integrin receptors which bind to these proteins. Of the fibronectin binding integrins, Capan1 and nRhoC cells showed minimal expression of integrin αvβ5, moderate amounts of integrin αvβ3, αvβ6, αvβ8 and a significantly high level of integrin α5β1 (Figure S5A). Over-expression of RhoC constructs (nRhoC, cRhoC, nDCT) did not alter the surface, or the total expression levels of these integrins (Figure S5B).

**Figure 5:**
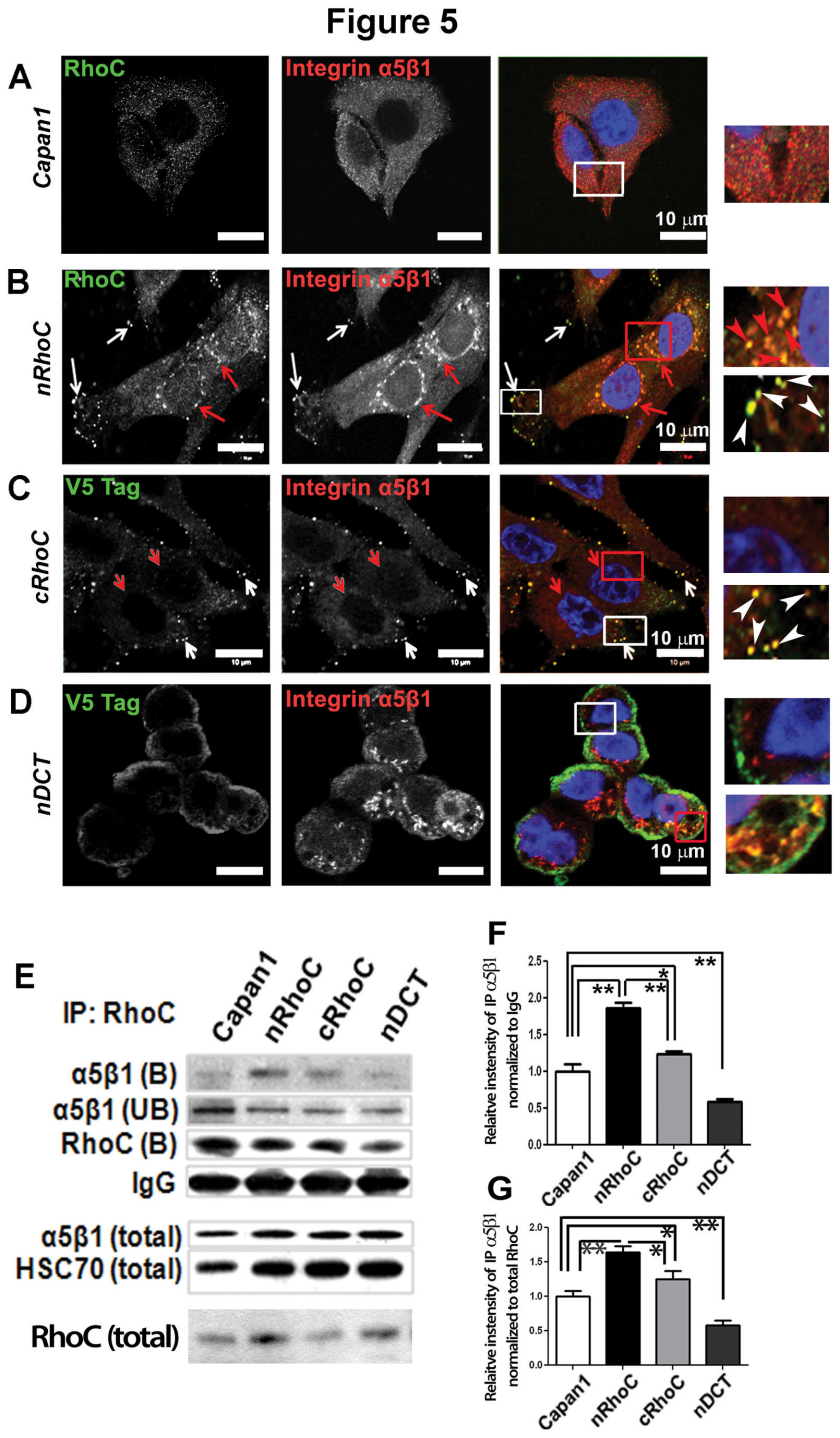
RhoC co-localized with integrin α5β1 at cell protrusions and in the peri-nuclear region. (A) In parental Capan1 cells there was minimal diffuse expression of RhoC (see also Figures S1, S2) and, similarly, diffuse expression of integrin α5β1 (Figures S3, S4). (B) In nRhoC cells, the exogenous RhoC co-localized with integrin α5β1 at a cell protrusion (white arrows and boxes) and in the peri-nuclear region (red arrows and boxes). (C) This was in contrast to cRhoC cells which showed co-localization of the V5 tag (cRhoC) with integrin α5β1 at the periphery of cells but not in the peri-nuclear region. (D) nDCT cells showed no co-localization of the V5 tag with integrin α5β1 at the plasma membrane (white box). In a few cells, diffuse cytoplasmic staining could be seen (red box). Scale bar: 10µm. This co-localization (confirmed by co-localization software in the confocal microscope) in distinct cellular compartments could be seen in 3D during Transwell migration (Figures S3, S4). Further explanation of the likely cellular compartment of this co-localization is provided in Figure S6. (E) Immuno-precipitation (IP) using anti-RhoC antibody (against C-terminal 100-193 amino acids) confirmed the interaction of RhoC and integrin α5β1. Densitometric quantification, when normalization was carried out for the immuno-precipitation reaction (IgG, F) or for the input (total RhoC, G), of the IP-bound integrin α5β1 showed significant increase of integrin α5β1 interaction with RhoC in both nRhoC and cRhoC cell lines. A significant reduction in this interaction with integrin α5β1 was observed in the nDCT cells as compared with parental cell lines. Appropriate IgG controls and reverse IP are shown in Figure S7. B:IP-bound fraction; UB: IP-unbound fraction; total: total lysate. *p<0.05, **p<0.01, Student’s t-test, error bars: SEM.

However, microscopic analysis revealed co-localization of RhoC and integrin α5β1 at the cell protrusion ends, as well as in the peri-nuclear regions in both 2D (Figure 5A-D) and 3D culture (Figures S3,S4,S6). This co-localization was confirmed as being the result of direct or indirect (in a complex) protein-protein interaction *in vivo*, as shown by protein immunoprecipitation in Capan1 protein extracts, using either anti-RhoC or anti-integrin α5β1 or anti-integrin α5 antibodies (Figures 4E, S7). Co-localization of integrin α5β1 with other Rho proteins was minimal (Figures S6,S7). In the cRhoC cells (masked C-terminus CAAX motif of the RhoC to disrupt its CAAX motif-dependent post-translational processing [33]), the co-localization of tagged cRhoC protein and integrin α5β1 was mainly observed at the cell periphery (protrusive end) but, interestingly, not in the peri-nuclear region (Figure 4C). Furthermore, we did not observe such co-localization in nDCT cells at the perinuclear or focal adhesion sites (truncated form of RhoC protein with the C-terminal divergent region (amino acids 180-193) deleted, Figure 2D,E). In some cells cytoplasmic accumulation of nDCT as assessed by V5 tag could be seen. This suggests that the effect was RhoC-structure specific and that the C-terminal of the protein is important for this interaction.

Concordantly, with immunoprecipitation analysis, we found increased interaction of integrin α5β1 with RhoC in both nRhoC and cRhoC cells, but not in nDCT cells (Figures 5E, S7), suggesting that the interaction of integrin α5β1 with RhoC was dependent on its intact C-terminus. This localization was seen partly in recycling endosomes as well as lysosomes (Figure S6). 

### RhoC over-expression enhanced integrin α5β1 trafficking

**Figure 6:**
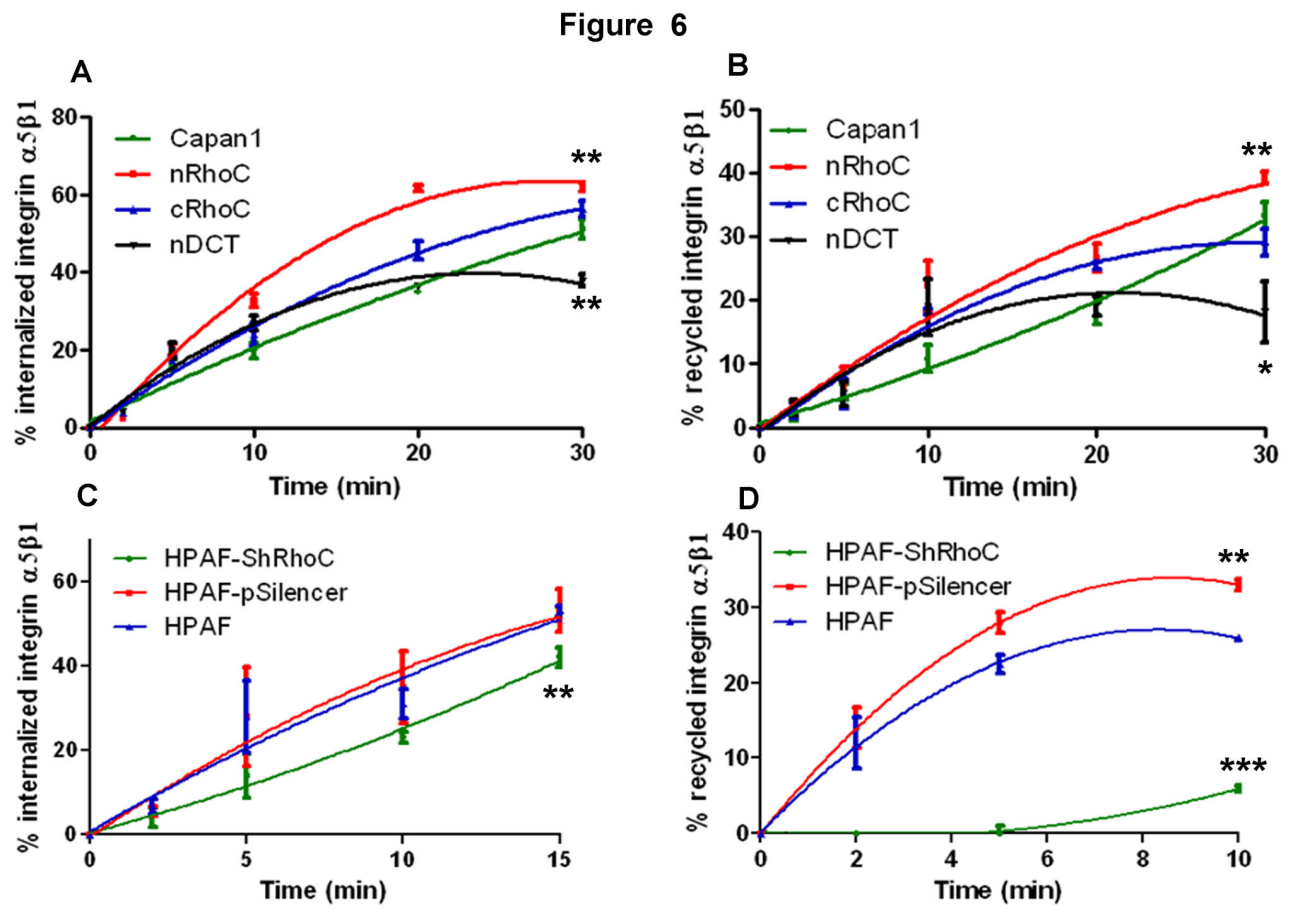
RhoC enhanced integrin α5β1 internalization and recycling upon fibronectin adherence. The well-established Biotin-labeling assay (labeling integrin α5β1 with Biotin and allowing internalization and recycling (separate assays: see Methods S1) followed by cleavage of Biotin and measurement of integrin by ELISA [18]) to compare the internalization and recycling rates of integrin α5β1. Graphs represent summary data from three representative individual experiments. The trend-line shown is second-order polynomial regression fit for the data, as previously used [46,47]. Thus, compared with parental Capan1 cells, nRhoC cells demonstrated significantly increased internalization (A) and recycling (B) of integrin α5β1 on fibronectin-coated surface. The masking of RhoC CAAX in cRhoC cells resulted in a significant increase in internalization, but not recycling, of integrin α5β1 (compared with parental Capan1 cells). However, deletion of the C-terminal of RhoC in nDCT cells resulted in significant reduction in internalization and recycling of integrin α5β1. Compared with the parental HPAF and pSilencer (empty vector) cells, shRhoC (stable RhoC knockdown) cells showed a significant decline of integrin α5β1 internalization (C) and recycling (D) on a fibronectin-coated surface. The dramatic reduction in the HPAF-shRhoC cells was not due to a vector artifact, since HPAF-pSilencer cells actually showed a significant enhancement of the recycling rate. Similar data were obtained after knock-down of endogenous RhoC in Panc0403 (not shown). In addition, there was no change in Transferrin receptor recycling after manipulation of RhoC (Figure S8). *** p<0.0001, ** p<0.001, * p<0.01, ANOVA. Error bars: SEM.

The distinct co-localization of RhoC and integrin α5β1 at cell protrusions and in the peri-nuclear region of nRhoC cells suggested that RhoC may be associated with integrin α5β1 intracellular trafficking which is known to follow ‘long-loop’ recycling from the cell surface to the peri-nuclear recycling compartment [34]. The standard Biotin-labeling assay [18], on fibronectin-coated surface, showed a significant increase in the nRhoC cells, compared with the parental cell line, in both the internalization and recycling rates of integrin α5β1 (Figure 6A, B). No difference was observed under non-coating conditions (data not shown). No change in Transferrin receptor recycling was observed (Figure S8)). Surprisingly, cRhoC cells showed enhanced internalization of integrin α5β1, but no alteration of the recycling rate; nDCT cells showed significantly diminished rates of both internalization and recycling of integrin α5β1 (Figure 6A, B), which we propose that it was due to lacking sufficient interaction between nDCT and integrin α5β1, and/or nDCT may also interfere the interaction of endogenous RhoC with integrin α5β1 [33,35]. Knocking down the endogenous RhoC expression in another two cancer cell lines HPAF (Figure 6C,D) and Panc0403 (data not shown) further confirmed the role of RhoC in the internalization and recycling of integrin α5β1. 

### Src activation is downstream of integrin α5β1

**Figure 7:**
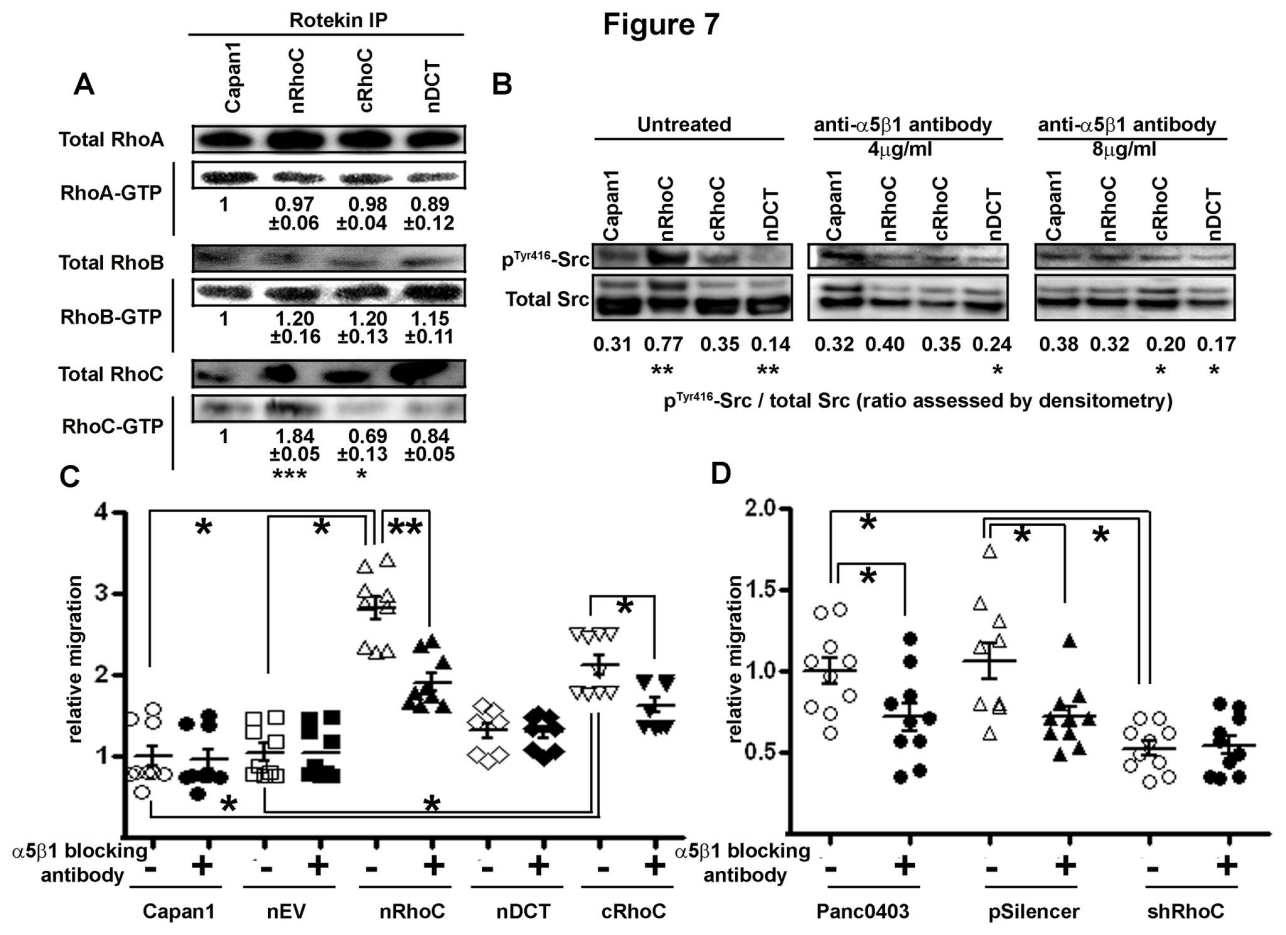
Src activation is downstream of RhoC-enhanced integrin α5β1 trafficking, partially contributing to increased cell migration. (A) Total RhoA and RhoB were unaltered upon transfection with various RhoC constructs in Capan1 cells. Rotekin immunoprecipitation binding assays revealed an increase of active RhoC-GTP in nRhoC cells (RhoA-GTP, RhoB-GTP levels were unaltered). Densitometric analysis for various RhoC-harboring constructs’ cell lines are normalized to parental Capan1 levels from triplicate experiments. (B) Upon plating on fibronectin, there was an increased level of phospho-Src (Tyr416) in nRhoC cells only. This increase was abrogated upon treatment with integrin α5β1-neutralizing antibody. Both cRhoC and nDCT cells showed significantly lower phospho-Src than parental Capan1 cells after treatment (IgG controls: no difference from the non-treated cells). (C) Functionally, both nRhoC and cRhoC cells showed enhanced migration in the IgG control conditions as compared with the parental, nEV, or nDCT cells. Blocking cells with 8μg/ml of the integrin α5β1-neutralizing antibody significantly diminished cell migration of both the nRhoC and cRhoC cells but did not alter basal migratory capacity of the nDCT cells. However, in nRhoC cells this reduction in migration capacity did not return to the level of parental or nEV cells. (D) Similarly in the endogenously high-expressing Panc0403 cells (also for HPAF (data not shown)) and the vector control transfected (pSilencer) cell line there was an abrogation of the enhanced migratory capacity upon blockade with integrin α5β1-neutralizing antibody compared with the lack of effect on the shRhoC cell line. *p<0.05, **p<0.001, Student’s t-test, error bars: SEM.

Recent evidence suggests an extensive crosstalk between integrins, the Src-family and small GTPases regulating a range of cellular processes [36–38]. Over-expression of the wild type RhoC (nRhoC) led to an increased amount of GTP-bound RhoC, with no changes in GTP-bound RhoA or RhoB (Figure 7A). Correspondingly, nRhoC cells, as compared with the parental cells, show a significant increase in phosphorylation at the Tyr416 residue of Src (no such increase was observed in cRhoC or nDCT cells, Figure 7B), as a measure of Src activation. This activation was reversed upon treatment with integrin α5β1-neutralizing antibody. In cRhoC cells, Src activation was suppressed below parental cell levels at 8μg/ml dose and in nDCT cells at both 4 and 8μg/ml doses. This implies that Src activation may be downstream of RhoC activation and its engagement with integrin α5β1. 

The enhanced migratory capacity as a result of RhoC overexpression (both nRhoC and cRhoC cells, Figure 2C), was reduced after integrin α5β1-neutralizing antibody treatment (8μg/ml). However, this intervention failed to restore the nRhoC-enhanced migration to the parental cell-line level, in spite of a near-complete abrogation of Src activation (Figure 7B, C). This suggests that RhoC may additionally promote cell migration through mechanism(s) independent of the integrin α5β1 trafficking-Src pathway, such as the recently investigated formin (FMNL1 [10] or FMNL3 [11]) recruitment at lamellipodial borders. Hence we did not further explore the effect of Src inhibition by agents such as Dasatinib, which in turn may affect multiple signalling cascades. Similar dependence upon an integrin α5β1 interaction with endogenous RhoC could be demonstrated in regulating the migratory potential of Panc0403 (Figures 7D). 

### RhoC and integrin α5β1 co-localization correlated with increased cell invasiveness in an organotypic culture model

**Figure 8:**
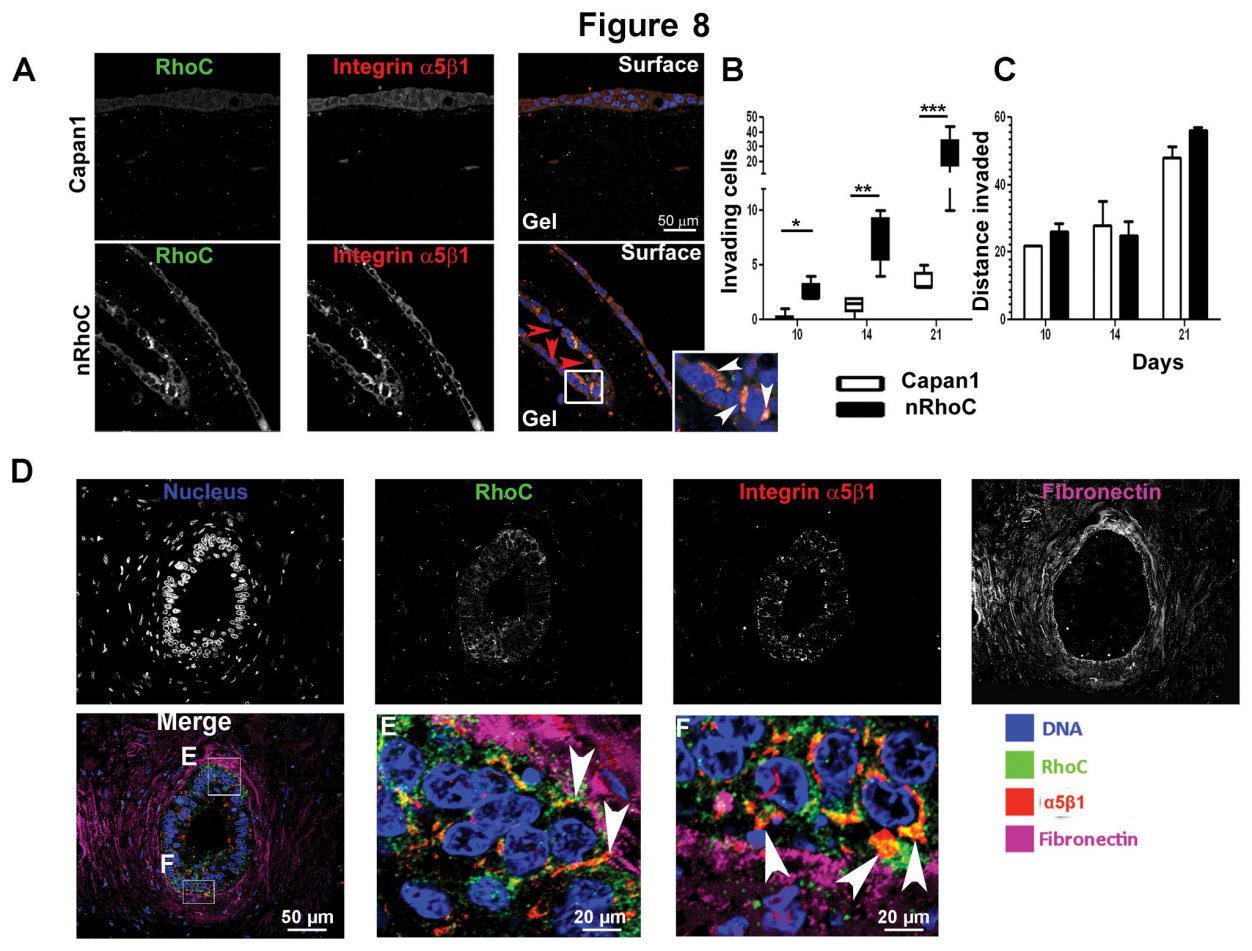
RhoC enhanced cell invasion into three-dimensional (3D) organotypic culture models. (A) Capan1 cells formed ductal structures on the surface of the gel in organotypic cultures, whereas the invading cells remained as individual cells within the gel. In contrast, invading nRhoC cells re-formed large ductal structures within the gel (red arrowheads within the lumen). Pronounced co-localization of RhoC and integrin α5β1 at the peri-nuclear region and cell protrusion end was observed particularly in invading nRhoC cells (inset). (B-C) Quantification of the total invading cell numbers (B), and average invading distance (C) at various time points confirmed a significant increase of invading nRhoC cell numbers compared with Capan1 cell numbers (though the invading distance was comparable). **p<0.001, Wilcoxon signed-rank test. (D)In human PDAC, co-localization of RhoC and integrin α5β1 peri-nuclear and cellular processes was seen in cancer cells (but not in stromal cells) where it was particularly prominent in those areas with increased peri-tumoral fibronectin (see merge picture and insets (E) and (F)). Scale bar 50 μm (20 μm for E and F).

In a validated three-dimensional organotypic culture system [21], nRhoC cells showed significantly greater numbers of invading cells compared with parental Capan1 cells (Figure 8A,B). Invaded nRhoC cells also formed ductal structures, whilst the invaded Capan1 cells still remained as individual cells, possibly reflecting, growth permissiveness under sub-optimal conditions, rendered by RhoC over-expression. Following up on this hypothesis, we show that while all cell lines grow at equal rates under optimal growth conditions (10% fetal bovine serum (FBS)), only nRhoC cells can maintain growth, albeit at a much slower rate, under sub-optimal conditions (3% FBS, Figure S9A). This effect could be related to our observation that Interleukin-6 secretion, but not growth factors (Figure S9B), was significantly enhanced in cells overexpressing wild-type, full length RhoC protein. Interleukin 6 was previously shown to mediate a survival benefit in squamous carcinoma cells [39,40]. 

Co-localization of RhoC and integrin α5β1, specifically the subcellular distribution, was more obvious in invading nRhoC cells (Figure 8A-C). Furthermore in human PDAC, there was significant upregulation of RhoC and integrin α5β1 in association with peri-tumoral fibronectin (Figure 8D-F, (fibronectin was minimal in organotypic cultures)). *In vitro* data suggest that caveolin-1 expression may negatively regulate RhoC-p38MAPK mediated migration in human pancreatic tumor cells [41]. In a different context, however, the disruption of caveolin-1 binding domain *in vitro* leads to the disruption of RhoC-mediated α5β1 integrin expression as well as Src activity and, thus, impairment of both migration and invasion [38]. In advanced pancreatic cancer of KPC mice we analyzed co-expression of RhoC, α5β1 integrin and caveolin1 *in vivo*. Interestingly, all three proteins show partial co-localization in epithelial tumor cells (Figure S10). However, we are unable to consistently observe this pattern throughout the majority of the tumor epithelium. Hence we did not pursue this investigation further. Nevertheless, the presence of caveolin-1 may indicate tumor cells with impaired capacity to migrate as previously suggested for human pancreatic tumor cells [41], or else its partial co-localization with RhoC and integrin a5β1 may be an indicator of cells with increased capacities for migration/invasion as suggested for melanoma and mammary epithelial tumor cells [38]. In a prostate cancer bone metastasis model, Collagen I attachment mediated by α2β1 integrin initiates motility programs through RhoC [42]. This suggests that the tumour micro-environment may provide necessary cues for RhoC driven changes. In addition to Collagen I, the stroma of PDAC is also rich in Collagen III and Fibronectin [43], which can bind to, and activate, α5β1 integrin. 

### RhoC and integrin α5β1 co-localization correlated with metastatic potential and poor differentiation status (transgenic murine model and human PDAC)

**Figure 9:**
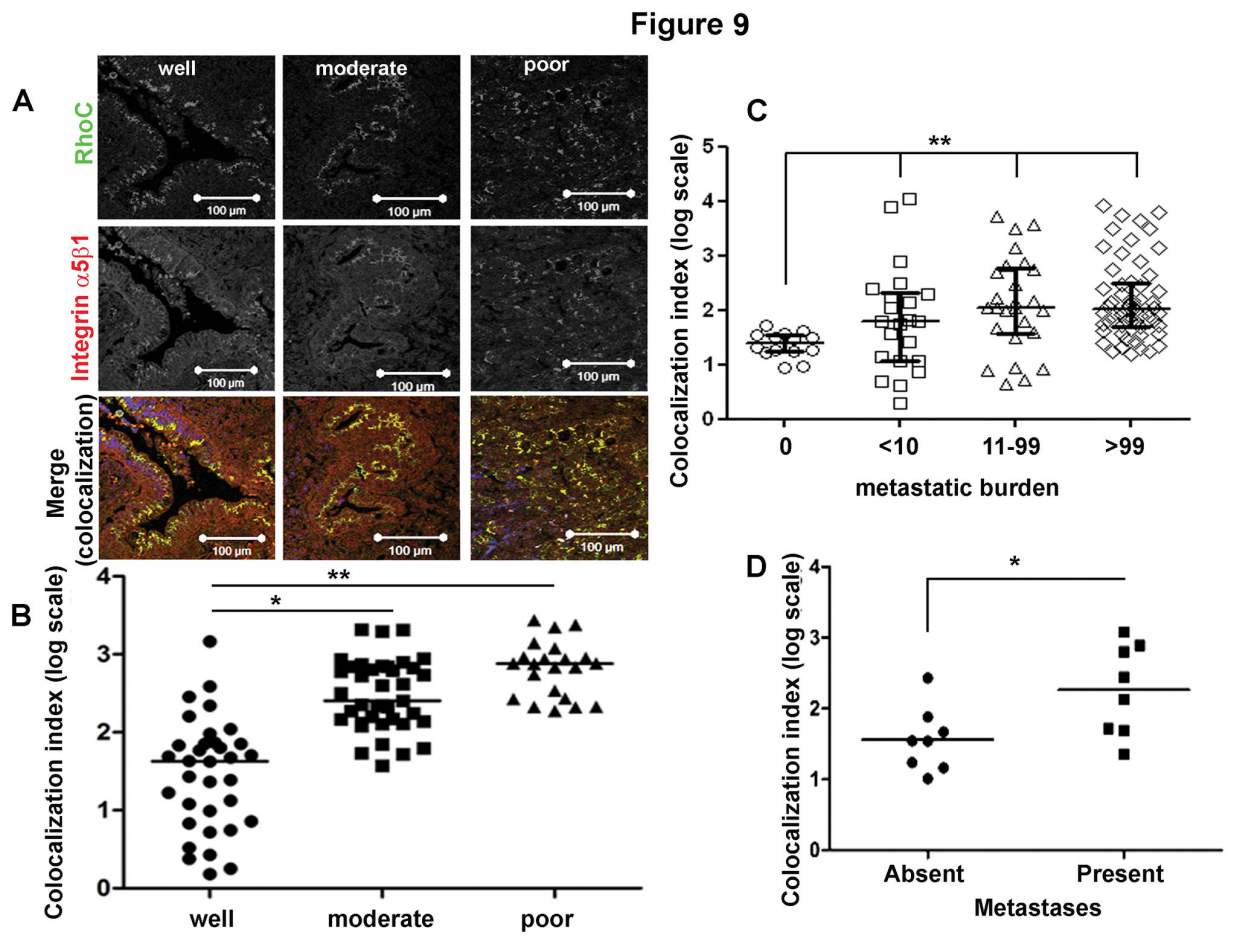
Co-localization of RhoC and integrin α5β1 correlated with poor differentiation status and metastatic potential in transgenic mouse and human PDAC lesions. Co-localization (index) was determined by independent observers using automated settings as described in Figure S11. The pathology grading /clinical data were not available to the scorers and the data were amalgamated independently to provide the correlations shown. All analyses were carried out in a blinded manner. The graphs represents a scatter plot with median value, Y axis is log- transformed co-localization index. (A) Co-localization of RhoC and integrin α5β1 correlated with the pathological differentiation level of human PDAC lesions. Increased co-localization levels were observed in the less differentiated human PDAC lesions. ((A), Red: integrin α5β1; Green: RhoC; Blue: DAPI; Merge: the co-localized sites in yellow.). (B) Co-localization Index was significantly higher in the moderately- (n=39) and poorly- (n=22) differentiated lesions in comparison with the well-differentiated (n=47) ones (*, p<0.05, **p<0.001, 1-way ANOVA test) in a cohort of patients where surgical resection was possible. (C) In another cohort of patients dying with PDAC (rapid autopsy programme, Johns Hopkins University) the co-localization index was significantly higher (**p=0.025, 1-way ANOVA test) in the group of patients dying with higher metastatic burden as compared with those with no metastasis at time of death. (D) Co-localization index was significantly higher (*p=0.02, Student’s t-test) in the primary cancer tissues in the group of KPC mice with distant metastases upon termination than in the group without distant metastases (see Figure S12).

In a large cohort of resected human PDAC samples, confocal microscopy imaging confirmed an increased ‘co-localization index’ (method of calculation shown in Figure S11) in moderate- (n=39) and poorly-differentiated (n=22) lesions in comparison with the well-differentiated (n=47) ones (Figure 9A, B). In another independent cohort of primary PDAC, obtained from a rapid autopsy programme for patients dying of PDAC [22], we found the co-localization index being significantly higher in patients with higher metastatic burden, compared with those with no metastases (Figure 9C), supporting the notion that the interaction of RhoC with integrin α5β1 is associated with tumor progression and metastatic proclivity.

Matched samples of primary and metastatic tumors from the same patients were not available to elucidate the functional importance of RhoC in the process of metastasis. Therefore, we examined the well-characterized transgenic mouse model (LSL-KrasG12D/+;LSL-Trp53R172H/+;Pdx-1-Cre, KPC mice, [23]) of PDAC, which develops invasive and highly metastatic PDA in a fashion that is very similar to that of human PDAs. In agreement with human *ex vivo* data, a similar tumor-grade dependent co-localization was observed in matched primary and metastatic tissue samples of KPC mice (Figure S12). Our survey of a small cohort of KPC mice found a significantly higher ‘co-localization index’ (P=0.02, n=8 each) in the group of mice with distant metastases upon termination than the ones without distant metastases (Figure 9D), indicating the vital functional role of RhoC in promoting tumor metastases in association with integrin α5β1 recycling. The importance of integrin recycling was also recently highlighted in a prostate cancer cell model where Trop-2, a trans-membrane glycoprotein, promoted metastatic dissemination by inducing the recycling and re-localization of integrin α5β1 from focal adhesions to the leading edges [44]. 

In this study, we highlight the role of RhoC (dependent on its C-terminus) in controlling the trafficking of integrin α5β1 in pancreatic cancer cells (see summary model in Figure S13), thereby enhancing cell migration and invasion; the major characteristics of the metastatic phenotype. We also demonstrate that the interaction of RhoC and integrin α5β1 is highly relevant in the poorly-differentiated lesions of PDAC; poor differentiation being a major determinant of poor prognosis and short survival of PDAC patients [45]. 

## Supporting Information

Methods S1 **Organotypic cultures, focal adhesion disassembly assay, Integrin internalization and recycling assays, Immunoprecipitation assays, Immunofluorescence.**
(DOC)

Table S1 **Results from LGC Standards Cell Line Authentication report are presented.**
(DOC)

Table S2 **Antibodies used for experiments.**
(DOC)

Table S3 **Primers used for cloning.**
(DOC)

Table S4 **shRhoC constructs.**
(DOC)

Figure S1 **Expression of endogenous Rho A/C as well as introduction of RhoC constructs in to pancreatic cancer cell lines.** (A) Endogenous protein expression levels of RhoC in normal (DEChTERT) and PDAC (Capan1: low, HPAF: high) cell lines as demonstrated along with HSC70 loading control.
(B) Endogenous protein expression levels of RhoA and RhoC PDAC cell lines as demonstrated along with HSC70 loading control. Bar graph represents the relative levels (densitometry results of triplicate Western blots when normalized to loading control HSC 70) of RhoA and RhoC expression in cancer cell lines. (error bars: SE).
(C) Schematic diagram of nRhoC (wild type) construct (full length RhoC cDNA with V5 and Lumio tags at the N-terminal (N)). Numbers indicate amino acid positions. SW1 and SWII are Switch I and II domains respectively where most of the effectors bind. The yellow area represents the terminal 13 amino acids which makes RhoC divergent from RhoA and RhoB and contains the CAAX motif.
(D) Schematic diagram of cRhoC (CAAX motif masked) construct (full length RhoC cDNA with V5 and Lumio tags at the C-terminus (C) to mask the CAAX motif).
(E) Schematic diagram of nDCT (C-terminal deleted) construct (with V5 and Lumio tags at the N-terminus).
(F) RT-PCR confirmed the mRNA expression of RhoC constructs in the stably transfected cell lines. See Table S3 for details of primers.
(PDF)

Figure S2 **Confirmation of alteration of RhoC expression in pancreatic cancer cell lines.** (A) ELISA analysis of V5 tag expression confirmed the expression of RhoC (V5) constructs. p64 line was Capan1 cells stably transfected with positive control plasmid of V5 and Lumio tags (Invitrogen), and thus was used as a positive control for V5 tag detection.
(B) Live cell labeling of Lumio-tag (red) confirmed the expression of RhoC constructs in live cells. The Lumio-red In-Cell-Labeling reagent was added into growth medium 30 minutes before live-imaging microscopy (Axiovert 200M microscope). Cells were maintained at 37°C in a humidified chamber. Capan1 parental cells were treated the same way to act as a negative control, and p64 cells were used as a positive control for Lumio-tag labeling. Scale bar: 10µm.
(C) Immunofluorescent staining of Capan1, nRhoC, cRhoC, nDCT and nEv cell lines with antibodies against V5 tag (green channel) and RhoC (Rabbit polyclonal anti-human C-terminal 100-193 amino acids, red channel) confirmed expression of transfected constructs as verified by imaging under confocal microscope (LSM 710, Carl Zeiss Inc.,). Pictures depict sub-cellular distribution along with marked changes in morphology such as flattened cells with spread cellular processes, especially, for nRhoC cells. Scale bar: 5µm.
(D-E) Bar graph represents the relative levels (densitometry results of triplicate Western blots when normalized to loading control HSC 70) of RhoC expression in Panc0403 cancer cell line after introduction of pSilencer (vector control) and shRhoC bearing pSilencer constructs. (**p<0.001, Student’s t-test, error bars: SE). Similar results were obtained for HPAF cells (data not shown).
(F) Comparative staining of endogenous RhoC in Panc0403, Panc0403-shRhoC and Panc0403-pSilencer lines using antibodies against RhoC C-terminal (G: Goat polyclonal anti-human RhoC, green channel) and C-terminal 100-193 amino acids (Rb: Rabbit polyclonal anti-human RhoC, red channel) confirmed the knockdown effect of RhoC protein in the Panc0403-shRhoC line. Images were acquired with Confocal microscope LSM 710 (Carl Zeiss Inc.,). Reproducible knockdown was achieved in HPAF cells similarly (data not shown). Scale bar: 5µm.
(PDF)

Figure S3 **RhoC expression in 3D in nRhoC cells.** (**A**) Confocal microscopy (LSM 710, Carl Zeiss Inc.,) Z stack images of the membrane of Transwell insert shows an increased RhoC expression in the migrated cells. The green, red and blue lines depict the cross sections along X, Y and Z axes respectively. The Z-stack XY image (is in the center, cross-section in Z plane by blue line) is on the migrated cells’ aspect demonstrating co-localization of RhoC and Integrin α5β1 (separate panels shown in panel B). The cross section along X-axis (green line, top panel: XZ plane) and Y-axis (red line, right hand panel: YZ plane) demonstrates migration of individual cells taking place across Transwell pores (demonstrated with broken white lines, two of the many pores demonstrated: see panel 9 of Figure C).
(**B**) In the nRhoC cells, increased RhoC expression was observed in conjunction with Integrin α5β1 expression in the peri-nuclear area (red arrowheads) and cell periphery (white arrowheads). These individual panels are from the XY face of the Figure A.
(**C**) The individual panels of the Z-stack (Figure A) are included to demonstrate uniformity of staining of the nucleus (DAPI) and show all the pores (*, middle panel: 9). Increased RhoC expression was observed in the migrated cells (panels 1-6) and low level of RhoC expression in the non-migrated cells on the opposite side (panels 13-18) of the Transwell insert (panels 7-12). Scale bar: 10µm.
(PDF)

Figure S4 **RhoC expression in 3D in Panc0403 cells.** (**A**) Staining of the Panc0403 line confirmed a significantly increased RhoC expression in the migrating cells and also displayed co-localization of RhoC with F-actin in the migrating cell body (Green: RhoC, Red: F-actin, Blue: DAPI) at the migrating-side of the membrane. Arrows highlight the cell body moving through a pore of the membrane (broken white lines). Non-migrated cells are shown in (B) The green, red and blue lines depict the cross section along X,Y and Z axes respectively. Images taken by Carl Zeiss LSM 710 microscope and Z-stack performed as shown. Alternative antibodies, as well as different staining and imaging methods, were used to rule out the possibility of staining/imaging artifacts (not shown). Scale bar: 10µm.
(PDF)

Figure S5 **FACS analysis for Integrin expression.** (A) FACS detection of surface expression of integrins on Capan1 and nRhoC cells showed no significant difference between these two lines. However, there was a significantly lower level of integrin αvβ3, αvβ6, αvβ8 and higher level of integrin α5β1 and α2 expression in both Capan1 and nRhoC lines.
(B) FACS detection of surface and total expression of integrin α5β1 in parental Capan1 line and the respective transfected cell lines did not show any significant differences.
(PDF)

Figure S6 **Co-localization of Rho GTPase and integrin α5β1.** (A) HPAF cells with high endogenous RhoC (green) demonstrate co-localization of integrin α5β1 the (red) in peri-nuclear region (white arrowheads in marked inset) and at cell protrusions (white arrowheads in main merge figure).
(B) In parental Capan1 cells, RhoB (red) (or RhoA (not shown)) expression was not co-localized with integrin α5β1 (green). See inset .
(C) nRhoC cells demonstrate partial co-localization of V5 (tagging RhoC) with endosomal marker (Early Endosomal Antigen 1: EEA1) at the perinuclear area (white arrowhead) and cell periphery (red arrowhead).
(D) nRhoC cells demonstrate partial co-localization of RhoC (green), V5 (tagging RhoC, red) with lysosomal marker (Lysosomal-associated membrane protein 1: LAMP1, blue) as shown by white arrowheads.
(E) nRhoC cells demonstrate partial co-localization of RhoC (green), V5 (tagging RhoC, red) with recycling endosomal marker (Rab11, blue) as shown by white arrowheads. Scale bar: 10µm.
(PDF)

Figure S7 **Immuno-precipitation.**
(A) Immuno-precipitation (IP) using anti-α5β1 antibody and probing for α5β1 (114 kD) revealed specificity of the IP method for parental Capan1 (lane 1) as well as derived cell lines: nRhoC (lane 2), cRhoC (lane 3), nDCT (lane 4), nEV (lane 5) in the immuno-precipitated fraction (IP#) and not in the unbound fraction (UB#). It also demonstrated the difficulty of reverse IP to demonstrate the bands for RhoC as the light chain IgG band (25kD) obscured the site.
(B) Further attempt at IP only the β1 subunit demonstrated that RhoA and RhoB (both MW ~ 18-22 kD) do not bind IP and are found only in the unbound fraction for the nRhoC cells.
(PDF)

Figure S8 **Transferrin recycling.** The well-established Biotin-labeling assay (labeling Transferrin Receptor with Biotin and allowing internalization followed by cleavage of Biotin and measurement of Transferrin Receptor by ELISA (6)) to compare the internalization rates of Transferrin Receptor. Graphs represent summary data from three representative individual experiments and the trend-line shown is second-order polynomial fit for the data. (A) Thus, compared to parental Capan1 cells, nRhoC, cRhoC and nDCT cells showed no significant change in internalization of Transferrin Receptor. (B) Similarly there was no change in shRhoC (stable RhoC knockdown) cells, compared to the parental HPAF and pSilencer (empty vector) cells on a fibronectin-coated surface. ANOVA. Error bars: SEM.
(PDF)

Figure S9 **Alteration in survival after overexpression of exogenous RhoC.** (A) Growth curve analysis of parental Capan1, nRhoC, cRhoC, nDCT and nEV cells showed no difference in population doublings under optimal culture conditions (10% FBS); however, under sub-optimal conditions (3% FBS, performed on day 40 onwards for Capan1, nEV and nRhoC cells) nRhoC cells sustained growth while parental Capan1 and nEV cells decreased population doublings dramatically. (**p<0.001, Student’s t-test, error bars: SE).
(B) Elisa (Mesoscale) analysis of supernatant demonstrates that nRhoC secretes high amounts of IL6 as compared to parental Capan1, nDCT, cRhoC cells or nEV (not shown). Other interleukins (IL1, IL2, IL8) and growth factors (VEGF (shown), EGF, FGF) showed no such change in expression upon transfection with various distinct RhoC constructs. (*p<0.01, Student’s t-test, error bars: SE). .
(PDF)

Figure S10 **Expression pattern of Caveolin-1 integrin a5β1 and RhoC in KPC mice tumors.** KPC mice tumors were stained with anti-caveolin-1, anti-integrin a5β1, and anti-RhoC antibodies. Partial co-localization of RhoC (purple) and caveolin-1 (red) can be seen in epithelial tumor cells. Co-localization of integrin a5β1 and RhoC in KPC mice tumors was almost universal. More detail in Figure S12 after quantification. Scale bar: 10 μm.
(PDF)

Figure S11 **Co-localization analysis of RhoC and integrin α5β1 using LSM710 Zen software.** The confocal image of a double-stained tissue section was loaded into Zen software in the ‘co-localization’ window. The Y-axis presents the RhoC staining intensity (green channel), and the X-axis presents the integrin α5β1 staining intensity (red channel). Only pixels that exist in both channels were plotted into the density plot. The images of normal pancreatic tissues were used to establish the base line defining the plot regions. Polygons were drawn to define the ROI (region of interest). Each ROI generated a density plot with region 1 (red channel above baseline), region 2 (the green channel above baseline) and region 3 (the overlap signals above baseline). Relevant data for each of the 1, 2 and 3 regions of each ROI were presented in the table of the analysis window. The data from region 3, the positive Co-localization region, were collected for calculating the ‘Co-localization Index’. Co-localization Index was calculated by multiplying region 3 data ‘Relative area (%)’, ‘Mean intensity ChS1-T2’ (RhoC mean intensity), ‘Weighted Coloc.Coefficient ChS1-T2’ (RhoC co-localization coefficient) and ‘Overlap Coefficient’ (RhoC and integrin α5β1 overlap coefficient). The index of normal tissue was zero since the baseline was set to ensure their ‘Relative area (%) of region 3’ was at zero level, and all malignant lesions were analyzed using the same baseline setting. Red squares highlighted the data used for the calculation of ‘Co-localization Index’.
(PDF)

Figure S12 **Co-localization of RhoC and integrin α5β1 correlated with poor differentiation status and enhanced metastatic potential in transgenic mouse pancreatic ductal adenocarcinoma (PDAC) lesions.** Increase in co-localization of RhoC and integrin α5β1 frequently was observed in poorly-differentiated tumors from the transgenic pancreatic cancer mouse model (LSL-KrasG12D/+;LSL-Trp53R172H/+;Pdx-1-Cre transgenic mice). This observation appeared more obvious in metastatic tumors rather than in primary ones. Metastatic lesions: well- and moderately-differentiated lesions were from liver metastasis, poorly-differentiated lesion was from lung metastasis (Green: RhoC; Red: integrin α5β1; Blue: DAPI; Merge: the co-localized sites in yellow). Scale bar: 20µm.
(PDF)

Figure S13 **Model of suggested RhoC-integrin α5β1-Src interactions.** RhoC interacts with integrin α5β1 and enhances its trafficking upon fibronectin adherence; this subsequently activates downstream Src. The interaction of RhoC and integrin α5β1 relies on the intact C-terminus divergent region of RhoC, while the translocation of the interacting RhoC- integrin α5β1 to the peri-nuclear region requires a CAAX motif-dependent, post-translational modification. Disruption of the internalization of integrin α5β1, by applying neutralizing antibody, or disruption of the recycling of integrin α5β1 by removal of CAAX motif-dependent membrane localization, abrogates the subsequent Src activation leading to a decrease in RhoC-enhanced cell migration.
(PDF)

Video S1 **Time-lapse imaging of cell adhesion and movement for Capan1 line.** The cells were imaged for the first two hours after plating on the fibronectin surface at 37°C in a humidified chamber using an Axiovert 200M microscope. Images were taken every 15 minutes and movie is played at 1 frame per second using the Simple PCI acquisition software.
(AVI)

Video S2 **Time-lapse imaging of cell adhesion and movement for nRhoC cell line.** The cells were imaged for the first two hours after plating on the fibronectin surface at 37°C in a humidified chamber using an Axiovert 200M microscope. Images were taken every 15 minutes and movie is played at 1 frame per second using the Simple PCI acquisition software.
(AVI)

Video S3 **Time-lapse imaging of cell adhesion and movement for nEV cell line.** The cells were imaged for the first two hours after plating on the fibronectin surface at 37°C in a humidified chamber using an Axiovert 200M microscope. Images were taken every 15 minutes and movie is played at 1 frame per second using the Simple PCI acquisition software.
(AVI)
