# Supplementary material for: RhoC Interacts with Integrin α5β1 and Enhances Its Trafficking in Migrating Pancreatic Carcinoma Cells
Source: PLoS One. 2013 Dec 3;8(12):e81575. doi: 10.1371/journal.pone.0081575 (PMC3849283; doi:10.1371/journal.pone.0081575)
Supplement: Methods S1 — Organotypic cultures, focal adhesion disassembly assay, Integrin internalization and recycling assays, Immunoprecipitation assays, Immunofluorescence. (DOC) [file pone.0081575.s001.doc]

# RhoC interacts with integrin α5β1 and enhances its trafficking in migrating pancreatic carcinoma cells

## Ningfeng Fiona Li^1*^, Emilios Gemenetzidis^1*^, Francis J Marshall^1*^, Derek Davies^2^, Yongwei Yu^3^, Kristopher Frese^4^, Fieke EM Froeling^1^, Adam K Woolf^1^, Roger M Feakins^5^, Yoshiki Naito^6^, Christine Iacobuzio-Donahue^6^, David A Tuveson^4^, Ian R Hart^1^, Hemant M Kocher^1, 5**^

^1^Barts Cancer Institute - a CR-UK Centre of Excellence, Queen Mary University of London, Centre for Tumour Biology, London, UK;

^2^Cancer Research UK London Research Institute, London, UK;

^3^Changhai Hospital of Shanghai Second Military Medical University, Pathology Department, Shanghai, China;

^4^Cancer Research UK Cambridge Research Institute, Cambridge, UK;

^5^Barts and the London HPB Centre, The Royal London Hospital, London, UK;

^6^Department of Pathology, The Johns Hopkins University School of Medicine, Baltimore, Maryland, USA.

* Contributed equally to this work

**Correspondence: [h.kocher@qmul.ac.uk](mailto:h.kocher@qmul.ac.uk), Centre for Tumour Biology, Barts Cancer Institute, London EC1M 6BQ

Tel: +44 20 7882 3579 Fax: +44 20 7882 3884

**Supplementary methods S1**

**Organotypic cultures:** In brief, Capan1 and nRhoC cells (or HPAF and shRhoC HPAF cells) were plated on the top of an organotypic gel system (combination of 75% Collagen type I and 25% Matrigel), submerged in complete growth medium as described before (1-3). Cultures were maintained for a pre-determined duration (10, 14 and 21 days) before harvesting and embedding in paraffin for sectioning for H&E- and immuno-staining, to determine the invasion index, expression of RhoC as well as any phenotypic changes.

**Focal adhesion disassembly assay** was used as described before (4, 5). Nocodazole was added into culture (cells plated on Fibronectin-coated coverslips and grown for three days and then in serum starved conditions (eight hours) prior to assay) at 10 µM for 15 minutes at 37^o^C to achieve maximum accumulation of focal adhesions and to depolymerize microtubules. FAK is required for microtubule-dependent focal adhesion disassembly (5). Cells were either fixed (stable focal adhesions) or further incubated in the serum-free medium for several intervals indicated in Figure 4C,D after washing-off Nocodazole, in order to determine focal adhesion kinetics. Quantification of mean paxillin staining intensity was done using LSM710 software on ~200 cells per time point, by two independent observers (NFL, HMK) in a blinded fashion (observers unaware of the treatment or cell type status). The paxillin intensity staining was performed at the cell periphery (site of focal adhesion formation) and the rest of the cell body was eliminated from analysis as shown in Figure 4C, D. The binary images, as demonstrated, allowed this well-validated assay to be carried out in an unbiased manner after acquisition of images. Data were assembled in GraphPad Prism5 software (La Jolla, CA, USA).

**Integrin internalization and recycling assays** Internalization and recycling assays were performed using well-validated methods described elsewhere (6). Cells were serum starved with or without fibronectin coating overnight. They were then transferred to ice and washed twice with ice cold PBS. Surface labeling was carried out with 2mg/ml NHS-SS-Biotin (Pierce, Rockford, USA) on ice for one hour at 4^o^C. Internalization of surface protein was allowed at 37^o^C, 5% CO_2_ for indicated time periods, and then non-internalized Biotin-labeling was removed by 20mM MesNa (sodium 2-sulfanylethanesulfonate) /50mM Tris (pH8.6) and 100mM NaCl treatment for 15 minutes on ice. MesNa solution was then quenched by addition of 20mM lodoacetamide for 10 minutes. Cells were lyzed. Biotinylated integrin α5β1 or Transferrin in lysates was determined using capture-ELISA. ELISA was carried out using 96-well plates coated with 5 μg/ml appropriate anti-integrin or anti-Transferrin antibodies (overnight) Na_2_CO_3_ at 4°C and blocked with 5% BSA in 0.05% Tween-20 in PBS (one hour at RT). 50 μl of cell lysate was left to incubate overnight at 4°C and unbound material was removed with PBS-T washes. Detection was done by streptavidin-conjugated horseradish peroxidase (1% BSA PBS-T containing, one hour, 4°C) and a chromogenic reaction with ortho-phenylenediamine.

For the recycling assay, surface labeled biotin was allowed to internalize for 30 minutes before removal of non-internalized biotin. Internalized biotinylated proteins were allowed to recycle back to the cell surface by incubating in 37^o^C, 5% CO_2_ for the indicated time period, followed by a second removal of surface-recycled biotin. The remaining integrin α5β1 was then determined by capture-ELISA assay.

**Immunoprecipitation assays**

Cell lysates were obtained using CHAPS buffer with protease inhibitors (Complete, Roche Diagnostics). Lysates were sonicated and insoluble material pelleted out. 150 μg of protein lysate was incubated overnight with pre-washed beads (40 μl anti-mouse or anti-rabbit IgG agarose, Sigma) and primary antibody (5 μg anti-α5β1 or anti- α5 or anti-RhoC/B/A) in combination. Controls were incubated with mouse or rabbit IgG1 (DAKO). After the first precipitation (centrifugation), the supernatant unbound fraction was separated (usually 900 μl), of which approximately 30 μl (~3%) was run as unbound fraction per gel. The remaining immunoprecipitate was washed 3 times with 1mL ice-cold PBS before the bead, with the bound proteins (approximately 20μl), was boiled with loading buffer for 10 minutes and ~7 μl loaded per gel (total ~15 μl). Both the immunoprecipitated and unbound fractions were run concurrently with both fractions being divided into two equal aliquots which could be probed with anti-RhoC and anti- α5β1 antibodies. Concurrent input Western blots from total cell lysates were run.

**Immunofluorescence** Cells on coverslips or Transwell inserts were fixed (4% formaldehyde/PBS) and permeabilized (0.2%Triton-X 100/PBS). Paraffin fixed tissue sections from KPC mice or patients were de-waxed and heated for 8 minutes in Citric Buffer (pH 6.0) for antigen retrieval. Antibodies (in 2%BSA/PBS) were incubated for 1 hour (room temperature) or 4^o^C (overnight). Images were captured using confocal microscopy (LSM710 Zen software, [Carl Zeiss Ltd., Welwyn Garden City](javascript:openContact('6C1847C9EBFC3A77C12570260031B700',true)), UK). Co-localization Index was calculated (Supplementary Figure 11) using manufacturer’s instructions by two observers (NFL, EG or HMK) independent of pathologic diagnosis (YY, RMF or CI-D). ‘Baseline zero’ was set up on relevant normal tissues.

**References:**

**1. Froeling FE, Feig C, Chelala C, Dobson R, Mein CE, Tuveson DA, et al. Retinoic Acid-Induced Pancreatic Stellate Cell Quiescence Reduces Paracrine Wnt-beta-Catenin Signaling to Slow Tumor Progression. Gastroenterology 2011;141:1486-97.**

**2. Froeling FE, Marshall JF, Kocher HM. Pancreatic cancer organotypic cultures. J Biotechnol 2010;148:16-23.**

**3. Froeling FE, Mirza TA, Feakins RM, Seedhar A, Elia G, Hart IR, et al. Organotypic culture model of pancreatic cancer demonstrates that stromal cells modulate E-cadherin, beta-catenin, and Ezrin expression in tumor cells. Am J Pathol 2009;175:636-48.**

**4. Jovic M, Naslavsky N, Rapaport D, Horowitz M, Caplan S. EHD1 regulates beta1 integrin endosomal transport: effects on focal adhesions, cell spreading and migration. J Cell Sci 2007;120:802-14.**

**5. Ezratty EJ, Partridge MA, Gundersen GG. Microtubule-induced focal adhesion disassembly is mediated by dynamin and focal adhesion kinase. Nat Cell Biol 2005;7:581-90.**

**6. Roberts M, Barry S, Woods A, van der Sluijs P, Norman J. PDGF-regulated rab4-dependent recycling of alphavbeta3 integrin from early endosomes is necessary for cell adhesion and spreading. Curr Biol 2001;11:1392-402.**
