# Supplementary material for: RhoC Interacts with Integrin α5β1 and Enhances Its Trafficking in Migrating Pancreatic Carcinoma Cells
Source: PLoS One. 2013 Dec 3;8(12):e81575. doi: 10.1371/journal.pone.0081575 (PMC3849283; doi:10.1371/journal.pone.0081575)
Supplement: Table S1 — Results from LGC Standards Cell Line Authentication report are presented. (DOC) [file pone.0081575.s002.doc]

| **Table S1: STR Profile of cell lines used for the experiments** | | | | | | |
| --- | --- | --- | --- | --- | --- | --- |
| **Powerplex16 Loci** | **ATCC reference** | **Customer sample** | **ATCC reference** | **Customer sample** | **ATCC reference** | **Customer sample** |
|  | **HTB-79** | **Capan1** | **CRL-2555** | **Panc-0403** | **CRL-1997** | **HPAF** |
| **AMELO** | X,X | X,X | X,X | X,X | X,X | X,X |
| **THO1** | 6,6 | 6,6 | 9.3,9.3 | 9.3,9.3 | 9,9 | 9,9 |
| **D5** | 11,11 | 11,11 | 11,11 | 11,11 | 11,13 | 11,13 |
| **D13** | 9,9 | 9,9 | 11,12 | 11,12 | 12,12 | 12,12 |
| **D7** | 10,11 | 10,11 | 8,12 | 8,12 | 10,13 | 10,13 |
| **D16** | 13,14 | 13,14 | 11,12 | 11,12 | 11,13 | 11,13 |
| **CSF** | 11,11 | 11,11 | 12,12 | 12,12 | 10,11 | 10,11 |
| **VWA** | 16,16 | 16,16 | 14,16 | 14,16 | 17,17 | 17,17 |
| **TPOX** | 8,11 | 8,11 | 10,11 | 10,11 | 8,8 | 8,8 |
