# Supplementary material for: RhoC Interacts with Integrin α5β1 and Enhances Its Trafficking in Migrating Pancreatic Carcinoma Cells
Source: PLoS One. 2013 Dec 3;8(12):e81575. doi: 10.1371/journal.pone.0081575 (PMC3849283; doi:10.1371/journal.pone.0081575)
Supplement: Table S2 — Antibodies used for experiments. (DOC) [file pone.0081575.s003.doc]

| **Table S2: Antibodies used for the experiments** | | | | |  |  |
| --- | --- | --- | --- | --- | --- | --- |
| **Primary antibody** | **Species raised in** | **Supplier** | **Dilution for IF** | **Dilution for WB** | **Dilution for FACS** | **Cell Culture** |
| α2 integrin | mouse | Chemicon (1950Z) | 1:100 |  | 5μg/ml |  |
| α4 integrin | mouse | Chemicon -7.2 | 1:100 |  | 5μg/ml |  |
| αvβ3 integrin | mouse | Chemicon (23C6) |  |  | 5μg/ml |  |
| αvβ5 integrin | mouse | Chemicon (P1F1) |  |  | 5μg/ml |  |
| αvβ6 integrin | mouse | Chemicon (10D5) |  |  | 5μg/ml |  |
| αvβ8 integrin | mouse | Chemicon (14E5) |  |  | 5μg/ml |  |
| α5 integrin | mouse | Chemicon -1969 | 1:100 |  |  |  |
| α5β1 integrin | rat | Millipore (JBS5) | 1:100 |  |  |  |
| α5β1 integrin (neutralizing) | mouse | Millipore |  |  |  | 4, 5 or 8μg/ml |
| HSC70 | mouse | Santa Cruz (sc-7298) |  | 1:5000 |  |  |
| RhoC (internal) | mouse | Abcam | 1:100 | 1:100 |  |  |
| RhoC (C-terminal 100-193aa) | rabbit | Abcam |  | 1:100 |  |  |
| RhoC (C-terminal) | Goat | Santa Cruz |  | 1:100 |  |  |
| V5 | rabbit | Abcam |  | 1:250 |  |  |
| Paxillin | mouse | Santa Cruz | 1:100 | 1:1000 |  |  |
| Src | rabbit | Cell Signalling (#2108) |  | 1:500 |  |  |
| p-Src (Tyr416) | rabbit | Cell Signalling (#2101) |  | 1:500 |  |  |
| Fibronectin | mouse | Sigma Aldrich (IST-4) | 1:50 |  |  |  |
| RhoB | goat | Abcam | 1:40 | 1:40 |  |  |
| RhoA | mouse | Abcam | 1:40 | 1:40 |  |  |
| EEA1 | rabbit | Santacruz (sc-33585) | 1:100 |  |  |  |
| Rab11 | mouse | Upstate (#05-853) | 1:100 |  |  |  |
| LAMP1 | rabbit | Abcam (ab-24170-100) | 1:100 |  |  |  |
| Caveolin-1 | rabbit | Santacruz (sc-894) | 1:100 |  |  |  |
| Anti-mouse HRP | Goat | Abcam (ab-5879-250) |  | 1:1,000 |  |  |
| Biotinylated anti-rabbit | Swine | DAKO (#E0353) | 1:500 |  |  |  |
| Anti-mouse-Alexa-488 | Goat | Invitrogen (#A11029) | 1:500 |  |  |  |
| Anti-mouse-Alexa-488 | Rabbit | Invitrogen (#A11059) | 1:500 |  |  |  |
| Anti-rabbit-Alexa-488 | Goat | Invitrogen (#A11034) | 1:500 |  |  |  |
| Anti-mouse-Alexa-546 | Goat | Invitrogen (#A11003 ) | 1:500 |  |  |  |
| Anti-mouse-Alexa-633 | Goat | Invitrogen | 1:500 |  |  |  |
