# Supplementary material for: RhoC Interacts with Integrin α5β1 and Enhances Its Trafficking in Migrating Pancreatic Carcinoma Cells
Source: PLoS One. 2013 Dec 3;8(12):e81575. doi: 10.1371/journal.pone.0081575 (PMC3849283; doi:10.1371/journal.pone.0081575)
Supplement: Table S3 — Primers used for cloning. (DOC) [file pone.0081575.s004.doc]

| **Table S3: Primers for cloning** | | | | |
| --- | --- | --- | --- | --- |
| Full length RhoC | RhoC Fr1: | 5’-CACCATGGCTGCAATCCGAAAG-3’ |  |  |
|  | RhoC Rev1: | 5’-GAGAATGGGACAGCCCCTCCGACG-3’ |  |  |
| C-terminal deleted RhoC (DCT) | RhoC Fr1: | 5’-CACCATGGCTGCAATCCGAAAG-3’ |  |  |
|  | D180-193 Rev: | 5’-GAGGCCAGCCCGAGTGGCCATCTC-3’ |  |  |
