# Supplementary material for: RhoC Interacts with Integrin α5β1 and Enhances Its Trafficking in Migrating Pancreatic Carcinoma Cells
Source: PLoS One. 2013 Dec 3;8(12):e81575. doi: 10.1371/journal.pone.0081575 (PMC3849283; doi:10.1371/journal.pone.0081575)
Supplement: Table S4 — shRhoC constructs. (DOC) [file pone.0081575.s005.doc]

| **Table S4: Constructs for shRhoC** | | | | |
| --- | --- | --- | --- | --- |
|  |  | 5’-AACATTCCTGAGAAGTGGACC-3’ |  |  |
|  |  | 5’-CGGATCAGTGCCTTTGGCTTGGCTA-3’ |  |  |
|  |  |  |  |  |
