# Supplementary figures and images for: RhoC Interacts with Integrin α5β1 and Enhances Its Trafficking in Migrating Pancreatic Carcinoma Cells
Source: PLoS One. 2013 Dec 3;8(12):e81575. doi: 10.1371/journal.pone.0081575 (PMC3849283; doi:10.1371/journal.pone.0081575)

# Supplementary Figure 1

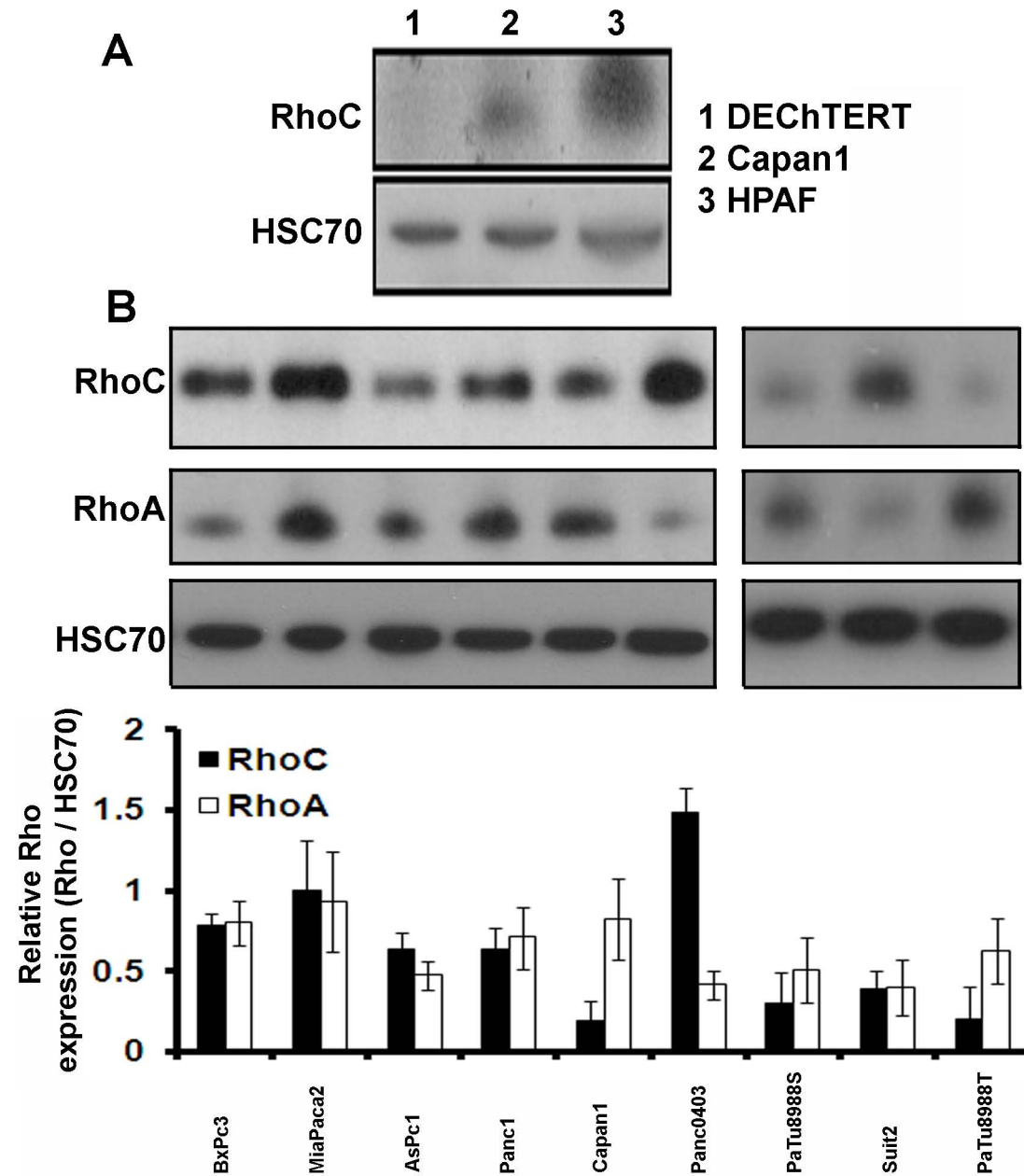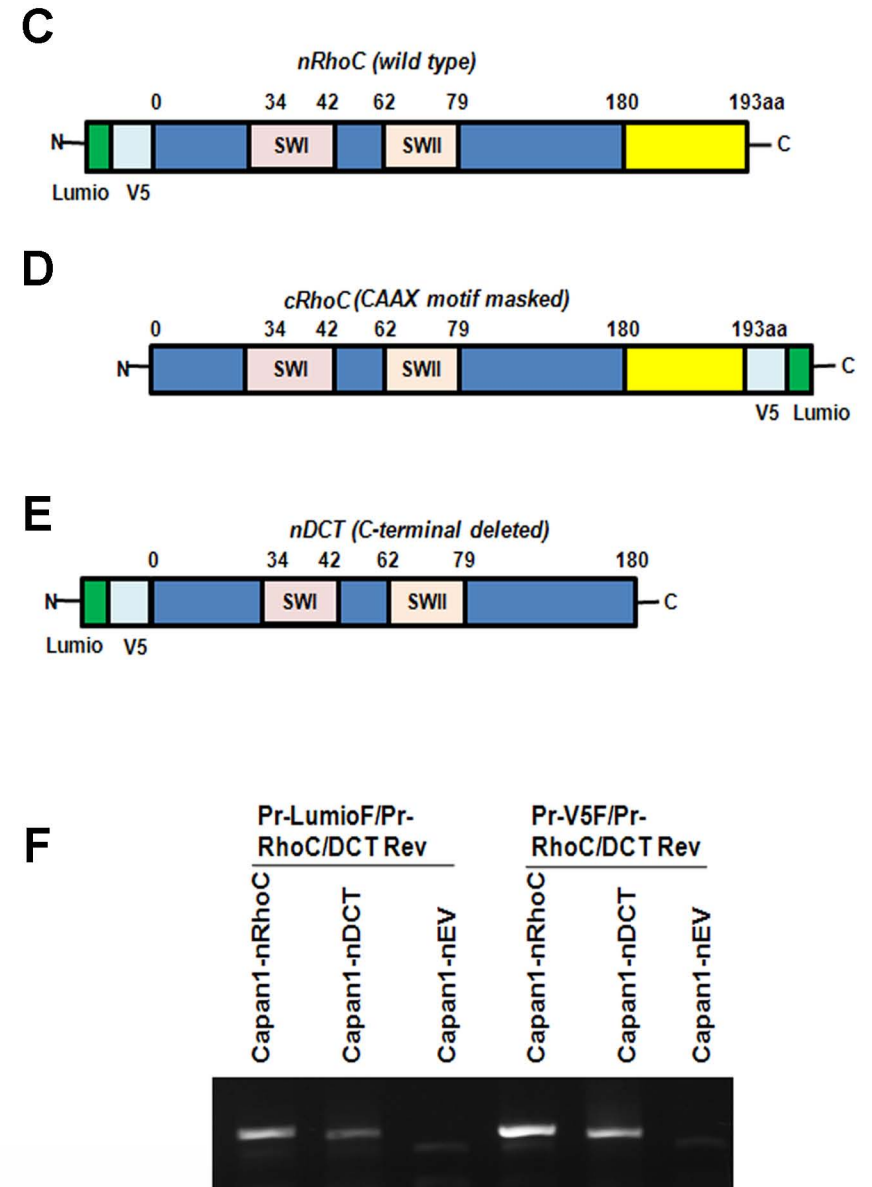

Supplement: Figure S1 — Expression of endogenous Rho A/C as well as introduction of RhoC constructs in to pancreatic cancer cell lines. (A) Endogenous protein expression levels of RhoC in normal (DEChTERT) and PDAC (Capan1: low, HPAF: high) cell lines as demonstrated along with HSC70 loading control. (B) Endogenous protein expression levels of RhoA and RhoC PDAC cell lines as demonstrated along with HSC70 loading control. Bar graph represents the relative levels (densitometry results of triplicate Western blots when normalized to loading control HSC 70) of RhoA and RhoC expression in cancer cell lines. (error bars: SE). (C) Schematic diagram of nRhoC (wild type) construct (full length RhoC cDNA with V5 and Lumio tags at the N-terminal (N)). Numbers indicate amino acid positions. SW1 and SWII are Switch I and II domains respectively where most of the effectors bind. The yellow area represents the terminal 13 amino acids which makes RhoC divergent from RhoA and RhoB and contains the CAAX motif. (D) Schematic diagram of cRhoC (CAAX motif masked) construct (full length RhoC cDNA with V5 and Lumio tags at the C-terminus (C) to mask the CAAX motif). (E) Schematic diagram of nDCT (C-terminal deleted) construct (with V5 and Lumio tags at the N-terminus). (F) RT-PCR confirmed the mRNA expression of RhoC constructs in the stably transfected cell lines. See Table S3 for details of primers. (PDF) [file pone.0081575.s006.pdf]

## Supplementary Figure 2

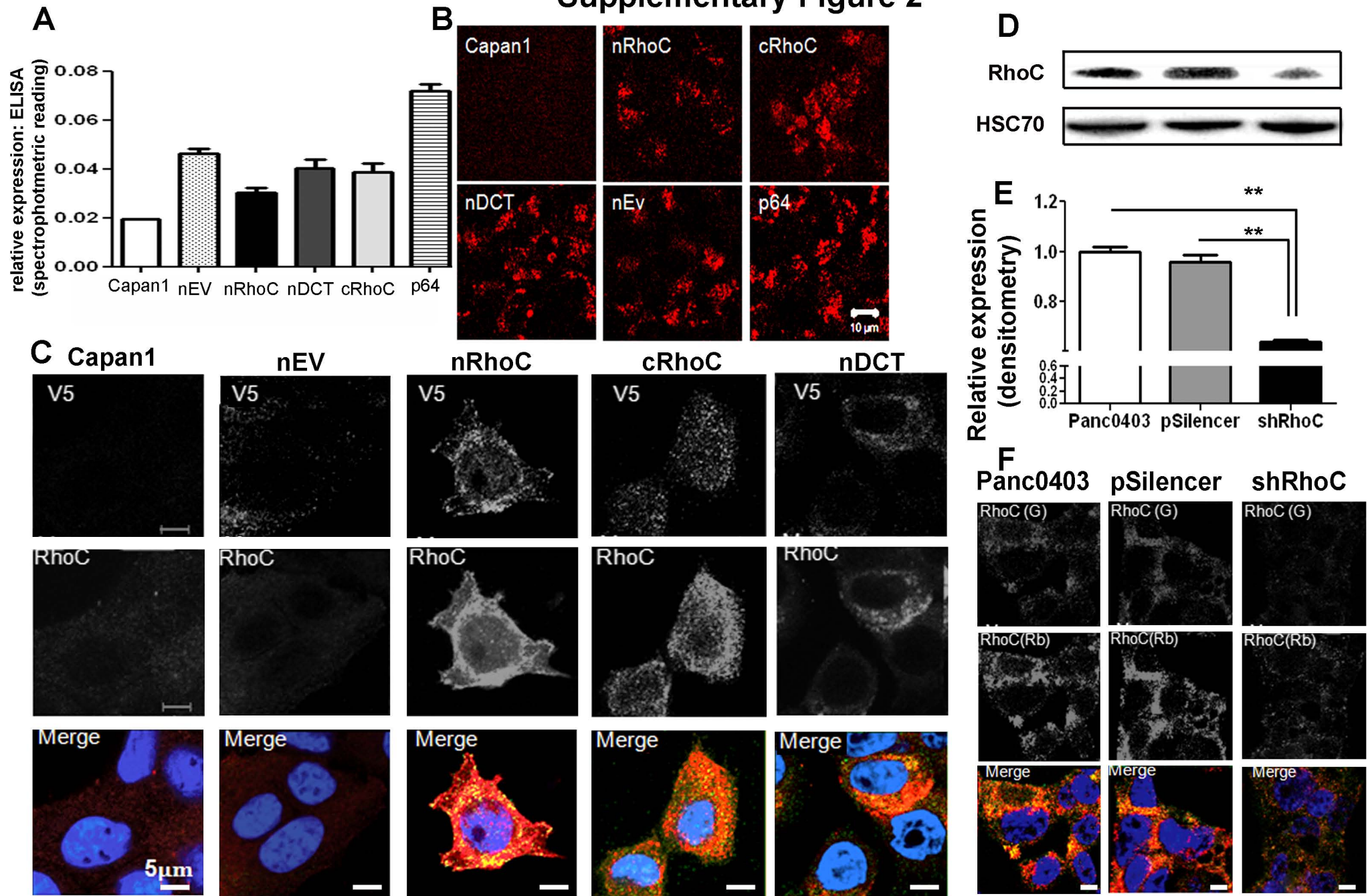

Supplement: Figure S2 — Confirmation of alteration of RhoC expression in pancreatic cancer cell lines. (A) ELISA analysis of V5 tag expression confirmed the expression of RhoC (V5) constructs. p64 line was Capan1 cells stably transfected with positive control plasmid of V5 and Lumio tags (Invitrogen), and thus was used as a positive control for V5 tag detection. (B) Live cell labeling of Lumio-tag (red) confirmed the expression of RhoC constructs in live cells. The Lumio-red In-Cell-Labeling reagent was added into growth medium 30 minutes before live-imaging microscopy (Axiovert 200M microscope). Cells were maintained at 37°C in a humidified chamber. Capan1 parental cells were treated the same way to act as a negative control, and p64 cells were used as a positive control for Lumio-tag labeling. Scale bar: 10µm. (C) Immunofluorescent staining of Capan1, nRhoC, cRhoC, nDCT and nEv cell lines with antibodies against V5 tag (green channel) and RhoC (Rabbit polyclonal anti-human C-terminal 100-193 amino acids, red channel) confirmed expression of transfected constructs as verified by imaging under confocal microscope (LSM 710, Carl Zeiss Inc.,). Pictures depict sub-cellular distribution along with marked changes in morphology such as flattened cells with spread cellular processes, especially, for nRhoC cells. Scale bar: 5µm. (D-E) Bar graph represents the relative levels (densitometry results of triplicate Western blots when normalized to loading control HSC 70) of RhoC expression in Panc0403 cancer cell line after introduction of pSilencer (vector control) and shRhoC bearing pSilencer constructs. (**p<0.001, Student’s t-test, error bars: SE). Similar results were obtained for HPAF cells (data not shown). (F) Comparative staining of endogenous RhoC in Panc0403, Panc0403-shRhoC and Panc0403-pSilencer lines using antibodies against RhoC C-terminal (G: Goat polyclonal anti-human RhoC, green channel) and C-terminal 100-193 amino acids (Rb: Rabbit polyclonal anti-human RhoC, red channel) confirmed [file pone.0081575.s007.pdf]

# Supplementary Figure 3

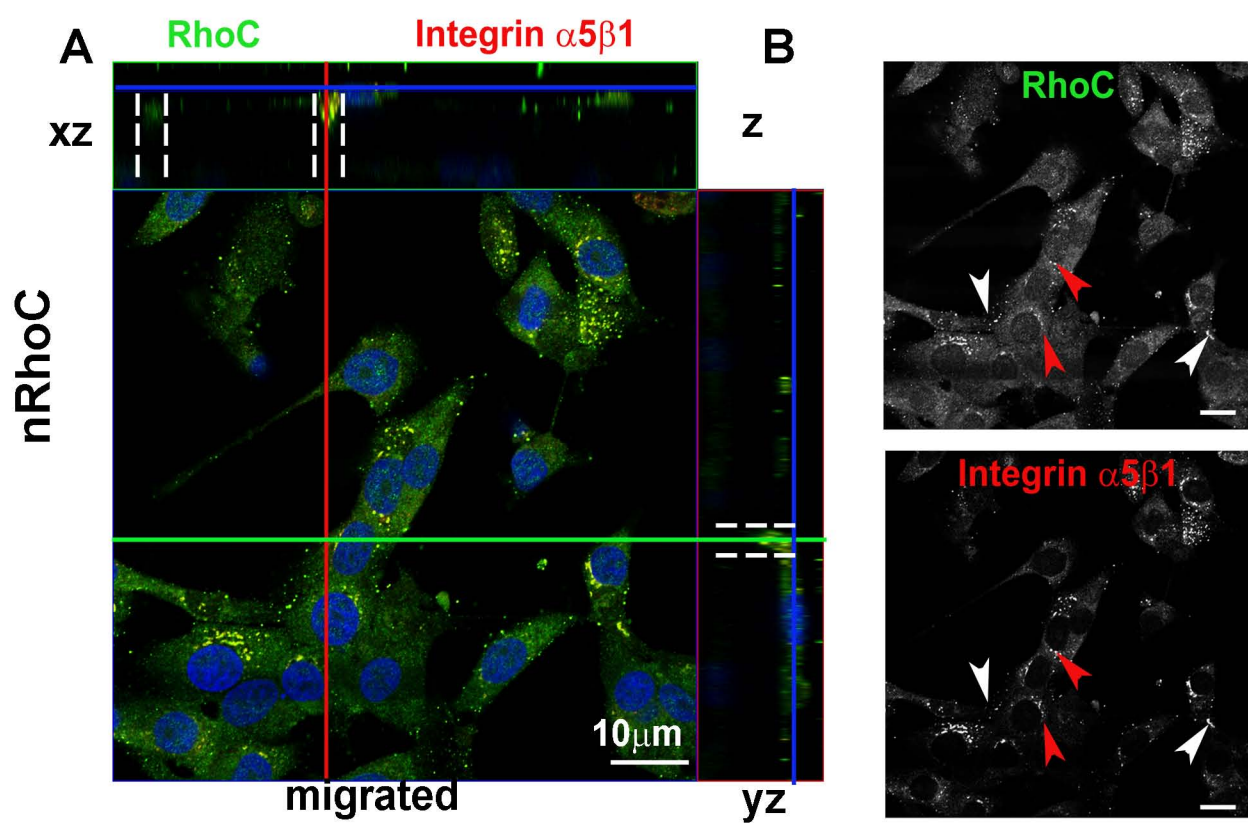

## C migrated

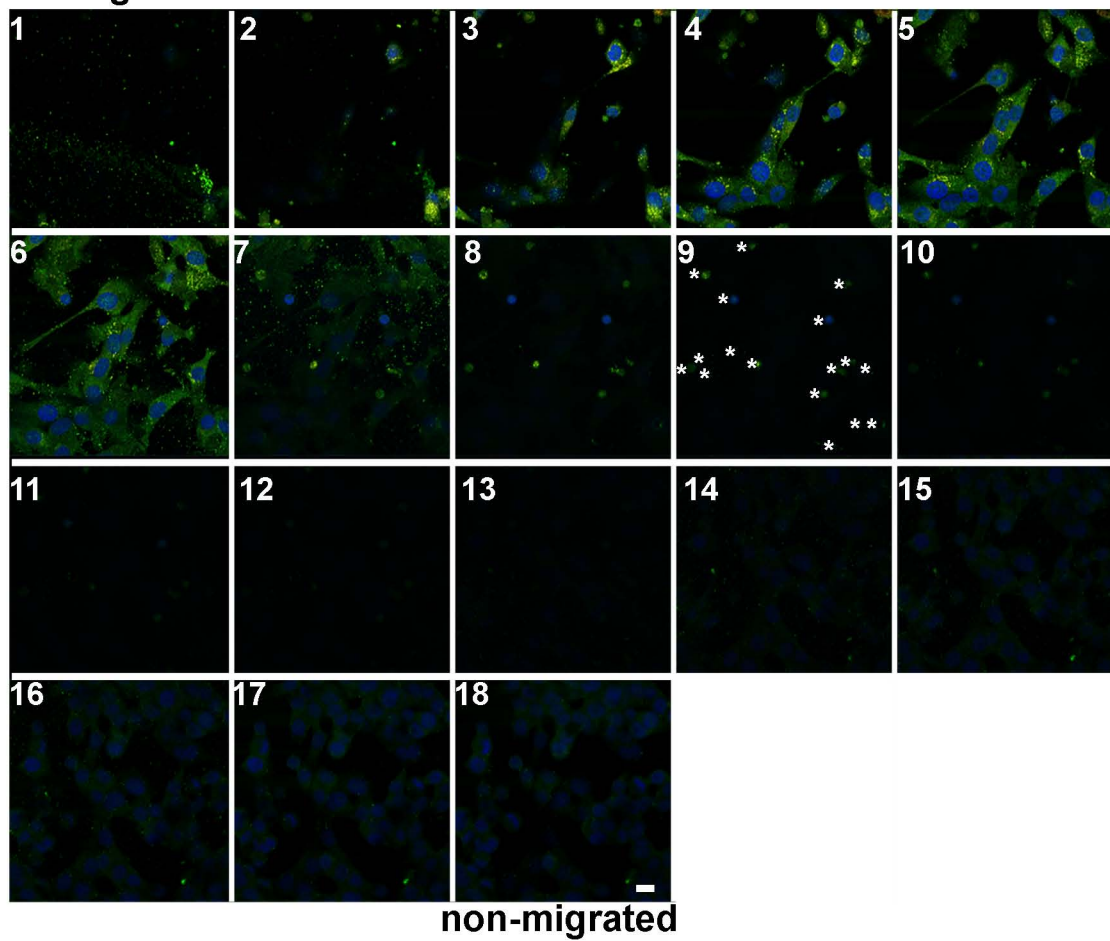

Supplement: Figure S3 — RhoC expression in 3D in nRhoC cells. (A) Confocal microscopy (LSM 710, Carl Zeiss Inc.,) Z stack images of the membrane of Transwell insert shows an increased RhoC expression in the migrated cells. The green, red and blue lines depict the cross sections along X, Y and Z axes respectively. The Z-stack XY image (is in the center, cross-section in Z plane by blue line) is on the migrated cells’ aspect demonstrating co-localization of RhoC and Integrin α5β1 (separate panels shown in panel B). The cross section along X-axis (green line, top panel: XZ plane) and Y-axis (red line, right hand panel: YZ plane) demonstrates migration of individual cells taking place across Transwell pores (demonstrated with broken white lines, two of the many pores demonstrated: see panel 9 of Figure C). (B) In the nRhoC cells, increased RhoC expression was observed in conjunction with Integrin α5β1 expression in the peri-nuclear area (red arrowheads) and cell periphery (white arrowheads). These individual panels are from the XY face of the Figure A. (C) The individual panels of the Z-stack (Figure A) are included to demonstrate uniformity of staining of the nucleus (DAPI) and show all the pores (*, middle panel: 9). Increased RhoC expression was observed in the migrated cells (panels 1-6) and low level of RhoC expression in the non-migrated cells on the opposite side (panels 13-18) of the Transwell insert (panels 7-12). Scale bar: 10µm. (PDF) [file pone.0081575.s008.pdf]

Supplementary Figure 4

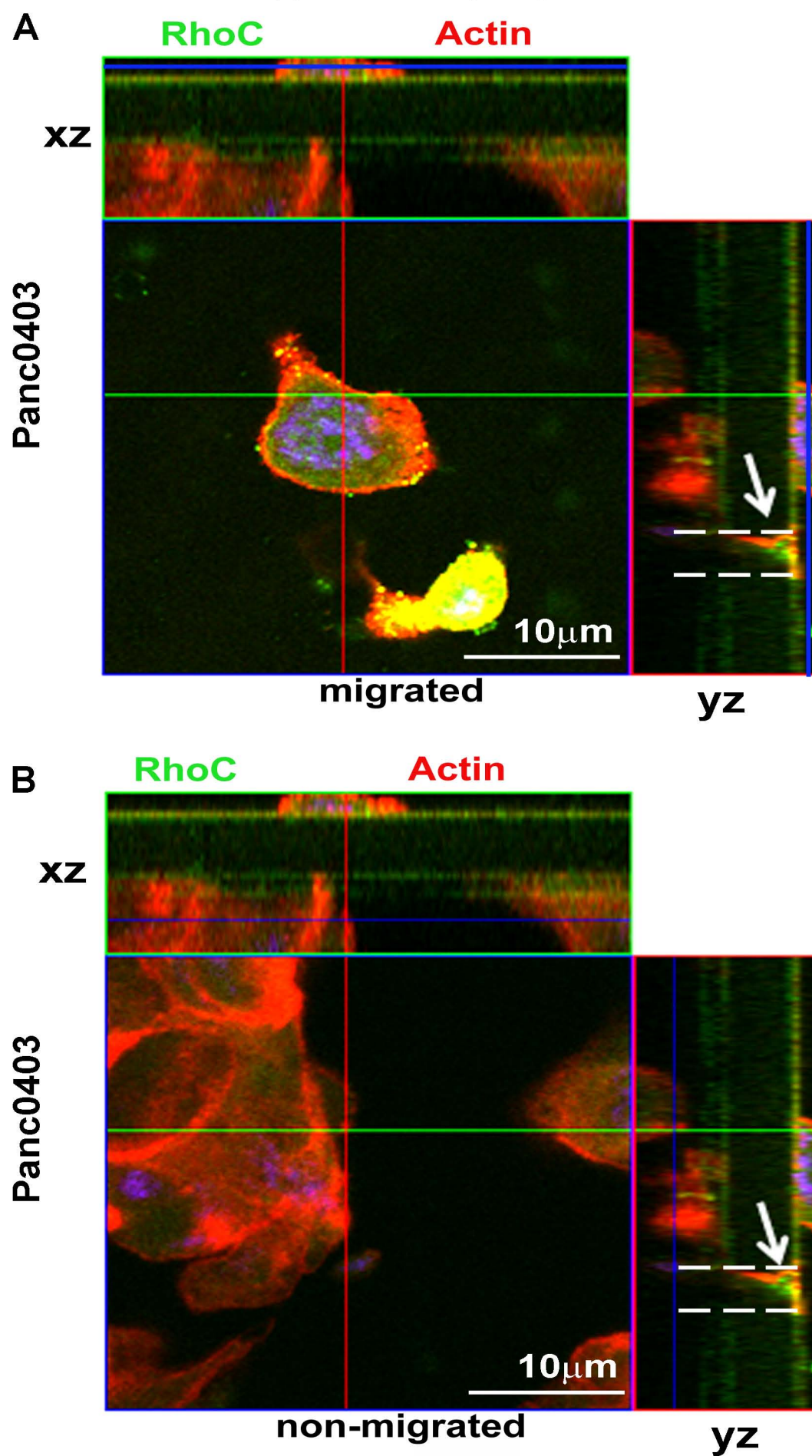

Supplement: Figure S4 — RhoC expression in 3D in Panc0403 cells. (A) Staining of the Panc0403 line confirmed a significantly increased RhoC expression in the migrating cells and also displayed co-localization of RhoC with F-actin in the migrating cell body (Green: RhoC, Red: F-actin, Blue: DAPI) at the migrating-side of the membrane. Arrows highlight the cell body moving through a pore of the membrane (broken white lines). Non-migrated cells are shown in (B) The green, red and blue lines depict the cross section along X,Y and Z axes respectively. Images taken by Carl Zeiss LSM 710 microscope and Z-stack performed as shown. Alternative antibodies, as well as different staining and imaging methods, were used to rule out the possibility of staining/imaging artifacts (not shown). Scale bar: 10µm. (PDF) [file pone.0081575.s009.pdf]

## Supplementary Figure 5

**A**

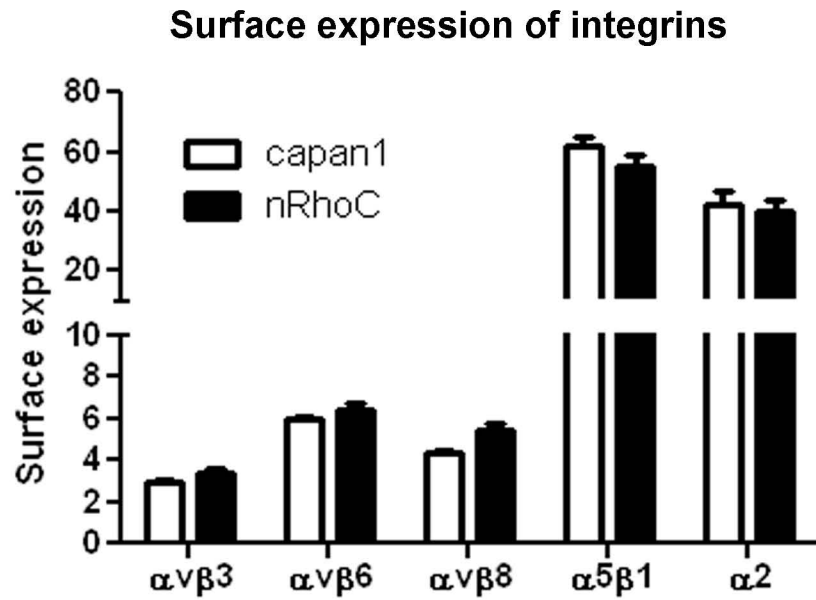

**B**

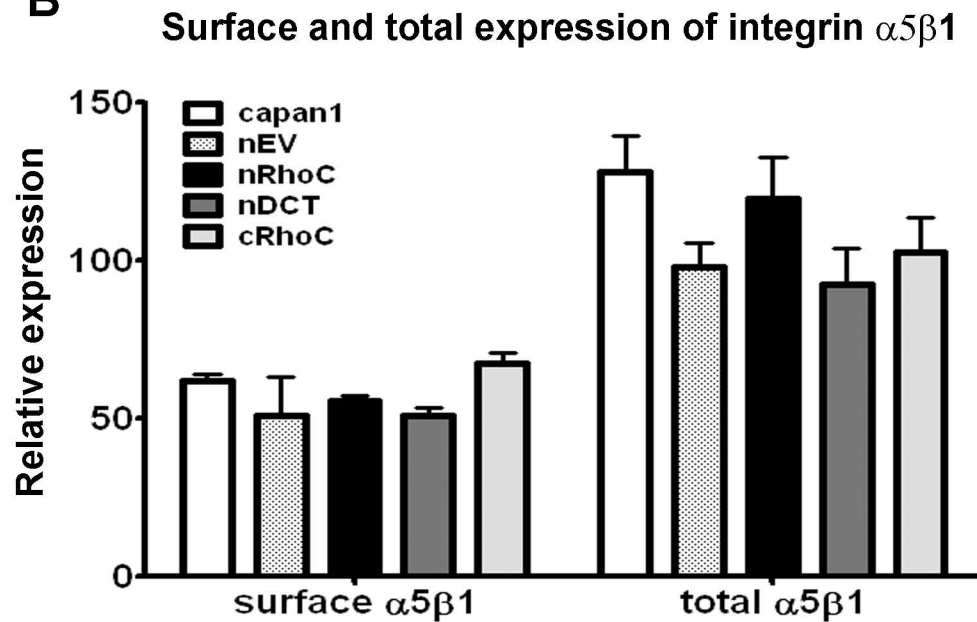

Supplement: Figure S5 — FACS analysis for Integrin expression. (A) FACS detection of surface expression of integrins on Capan1 and nRhoC cells showed no significant difference between these two lines. However, there was a significantly lower level of integrin αvβ3, αvβ6, αvβ8 and higher level of integrin α5β1 and α2 expression in both Capan1 and nRhoC lines. (B) FACS detection of surface and total expression of integrin α5β1 in parental Capan1 line and the respective transfected cell lines did not show any significant differences. (PDF) [file pone.0081575.s010.pdf]

# Supplementary Figure 6

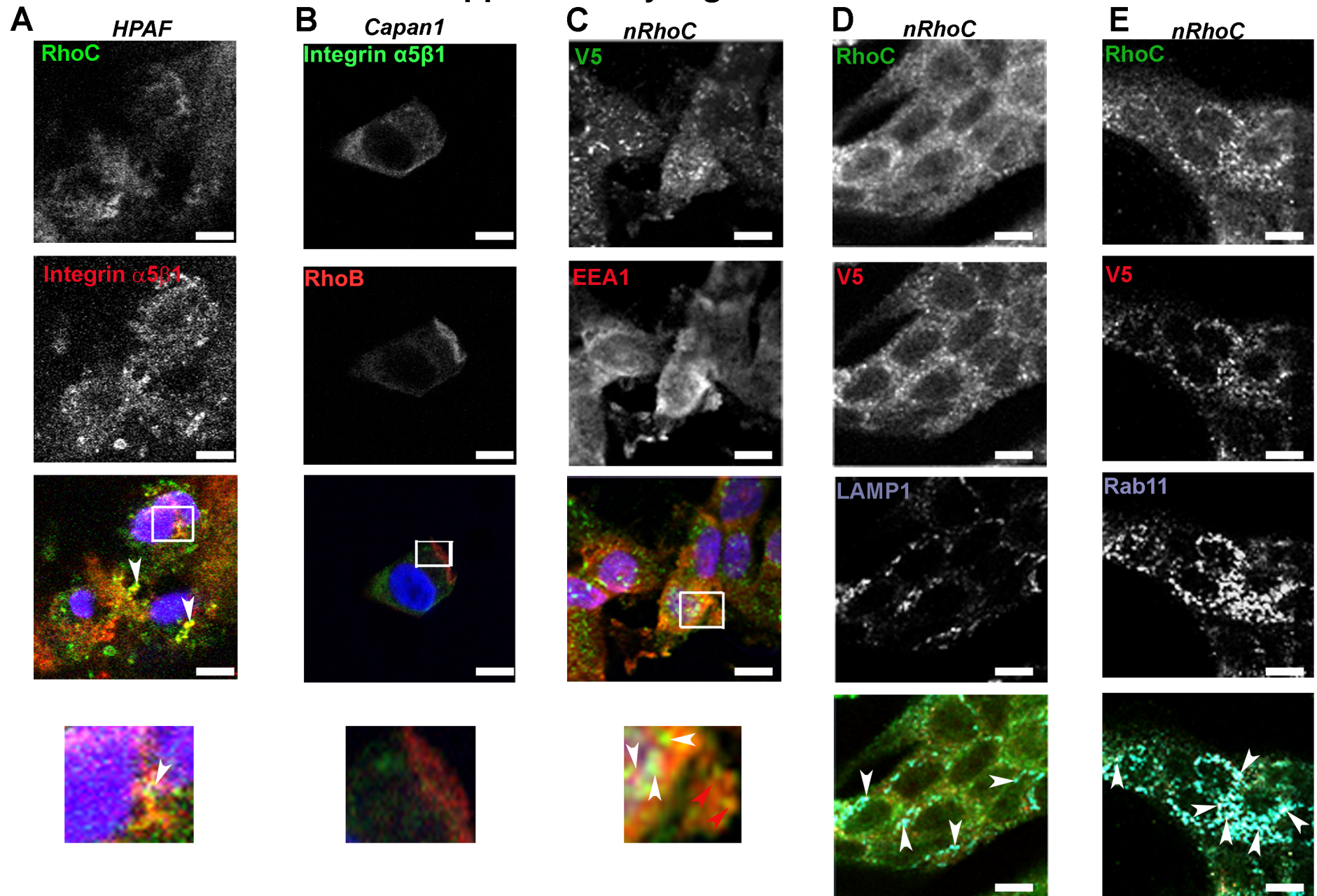

Supplement: Figure S6 — Co-localization of Rho GTPase and integrin α5β1. (A) HPAF cells with high endogenous RhoC (green) demonstrate co-localization of integrin α5β1 the (red) in peri-nuclear region (white arrowheads in marked inset) and at cell protrusions (white arrowheads in main merge figure). (B) In parental Capan1 cells, RhoB (red) (or RhoA (not shown)) expression was not co-localized with integrin α5β1 (green). See inset . (C) nRhoC cells demonstrate partial co-localization of V5 (tagging RhoC) with endosomal marker (Early Endosomal Antigen 1: EEA1) at the perinuclear area (white arrowhead) and cell periphery (red arrowhead). (D) nRhoC cells demonstrate partial co-localization of RhoC (green), V5 (tagging RhoC, red) with lysosomal marker (Lysosomal-associated membrane protein 1: LAMP1, blue) as shown by white arrowheads. (E) nRhoC cells demonstrate partial co-localization of RhoC (green), V5 (tagging RhoC, red) with recycling endosomal marker (Rab11, blue) as shown by white arrowheads. Scale bar: 10µm. (PDF) [file pone.0081575.s011.pdf]

## Supplementary Figure 7

**A**

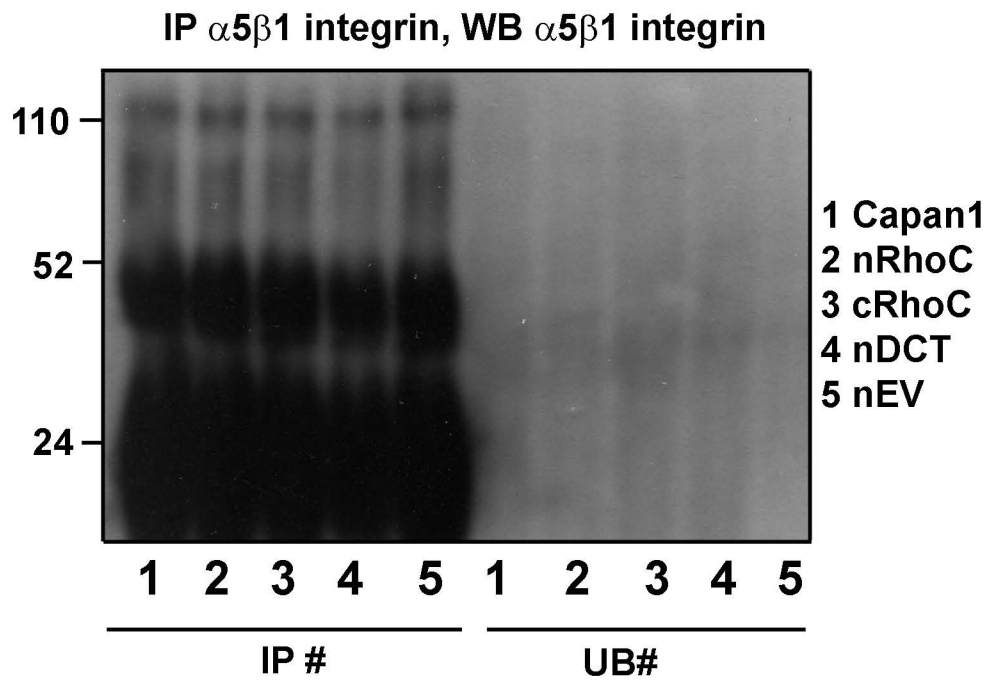

**B**

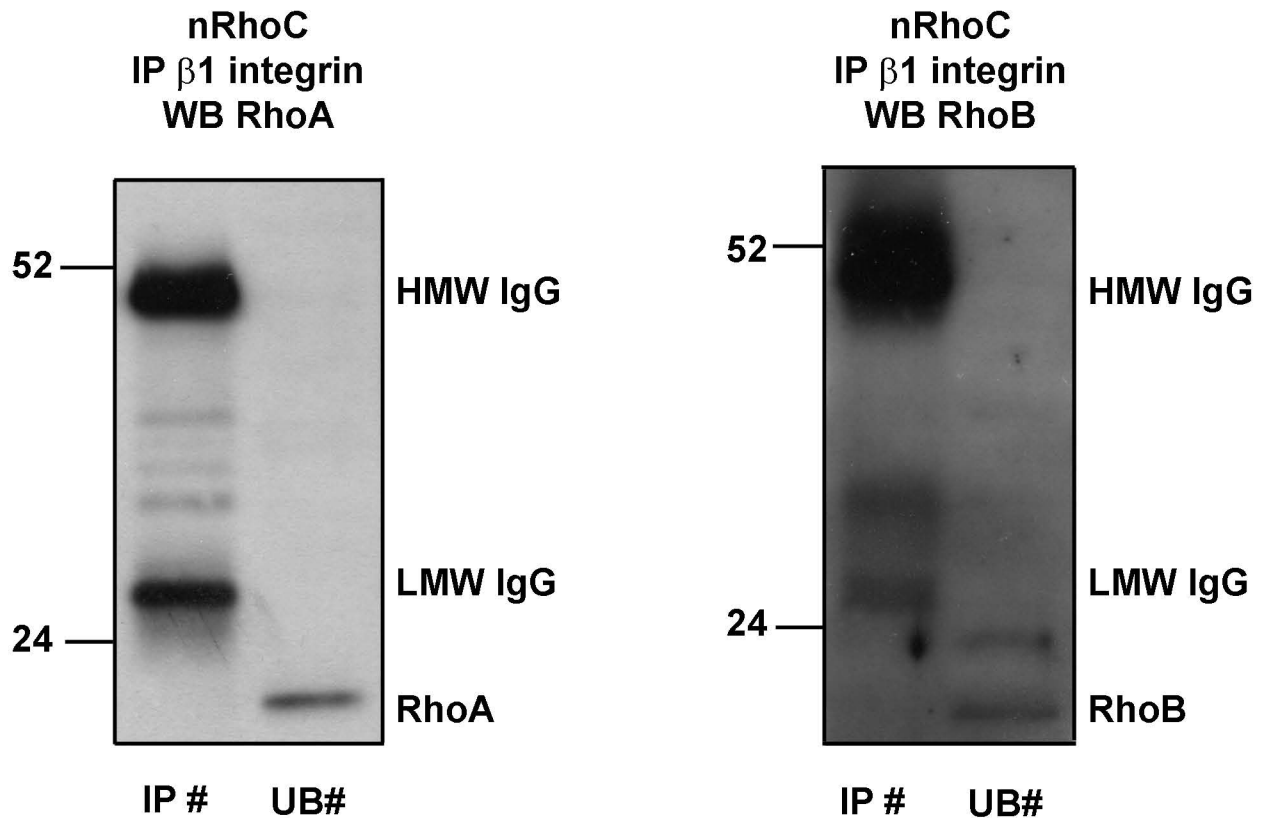

Supplement: Figure S7 — Immuno-precipitation. (A) Immuno-precipitation (IP) using anti-α5β1 antibody and probing for α5β1 (114 kD) revealed specificity of the IP method for parental Capan1 (lane 1) as well as derived cell lines: nRhoC (lane 2), cRhoC (lane 3), nDCT (lane 4), nEV (lane 5) in the immuno-precipitated fraction (IP#) and not in the unbound fraction (UB#). It also demonstrated the difficulty of reverse IP to demonstrate the bands for RhoC as the light chain IgG band (25kD) obscured the site. (B) Further attempt at IP only the β1 subunit demonstrated that RhoA and RhoB (both MW ~ 18-22 kD) do not bind IP and are found only in the unbound fraction for the nRhoC cells. (PDF) [file pone.0081575.s012.pdf]

## Supplementary Figure 8

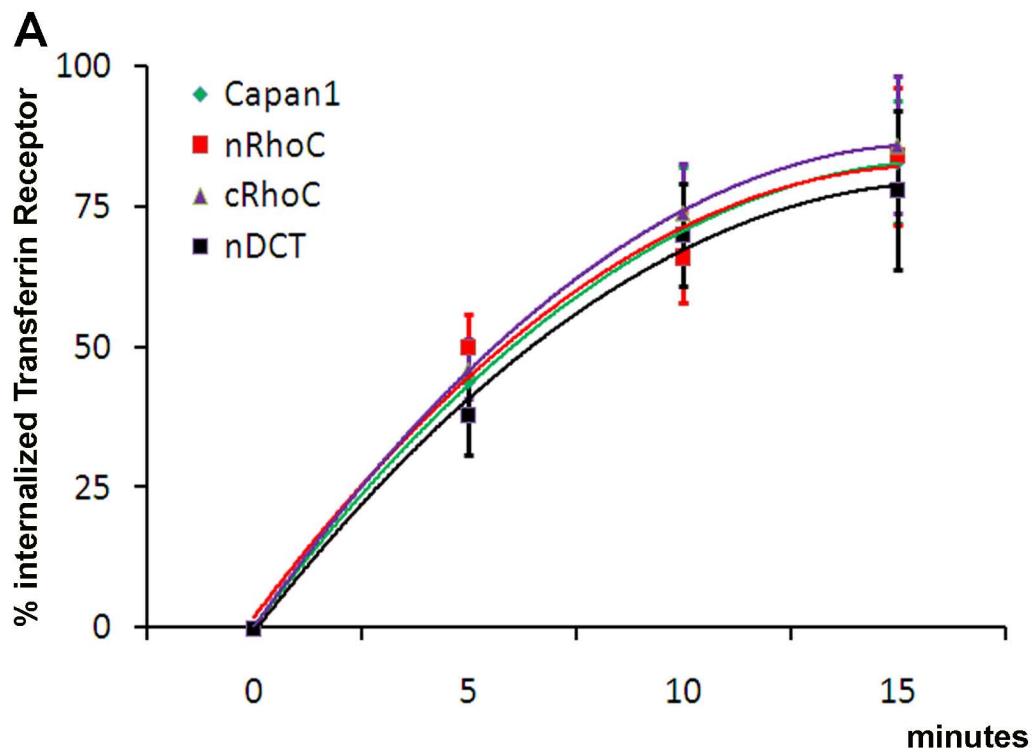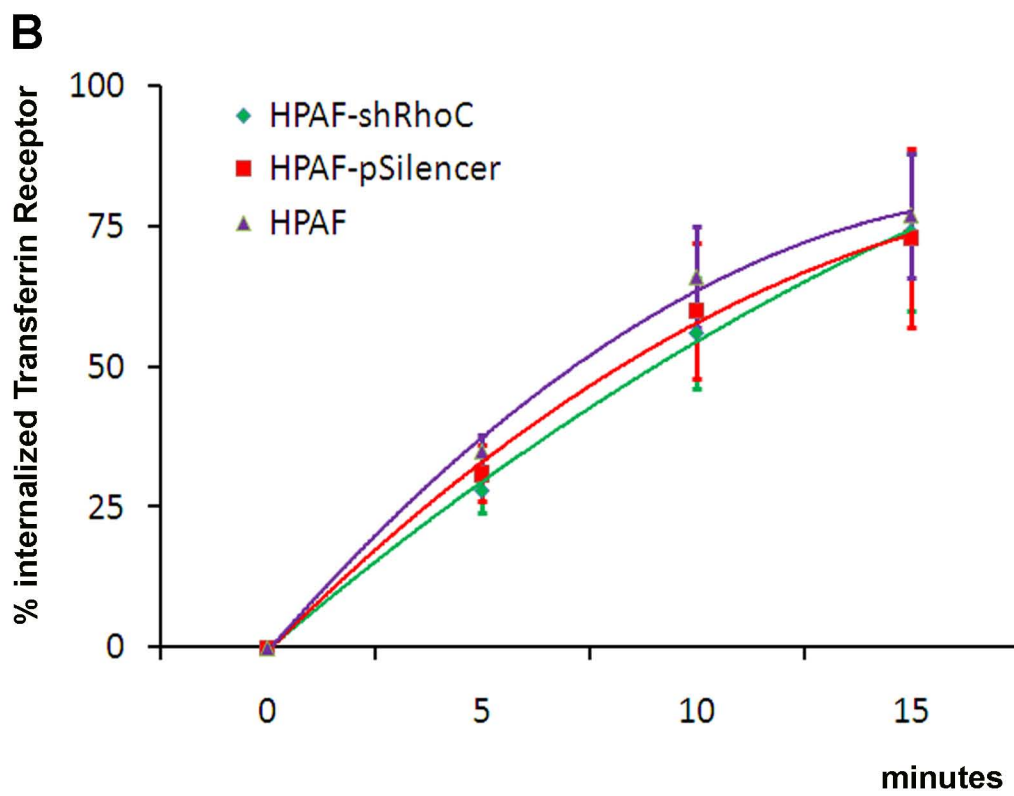

Supplement: Figure S8 — Transferrin recycling. The well-established Biotin-labeling assay (labeling Transferrin Receptor with Biotin and allowing internalization followed by cleavage of Biotin and measurement of Transferrin Receptor by ELISA (6)) to compare the internalization rates of Transferrin Receptor. Graphs represent summary data from three representative individual experiments and the trend-line shown is second-order polynomial fit for the data. (A) Thus, compared to parental Capan1 cells, nRhoC, cRhoC and nDCT cells showed no significant change in internalization of Transferrin Receptor. (B) Similarly there was no change in shRhoC (stable RhoC knockdown) cells, compared to the parental HPAF and pSilencer (empty vector) cells on a fibronectin-coated surface. ANOVA. Error bars: SEM. (PDF) [file pone.0081575.s013.pdf]

## Supplementary Figure 9

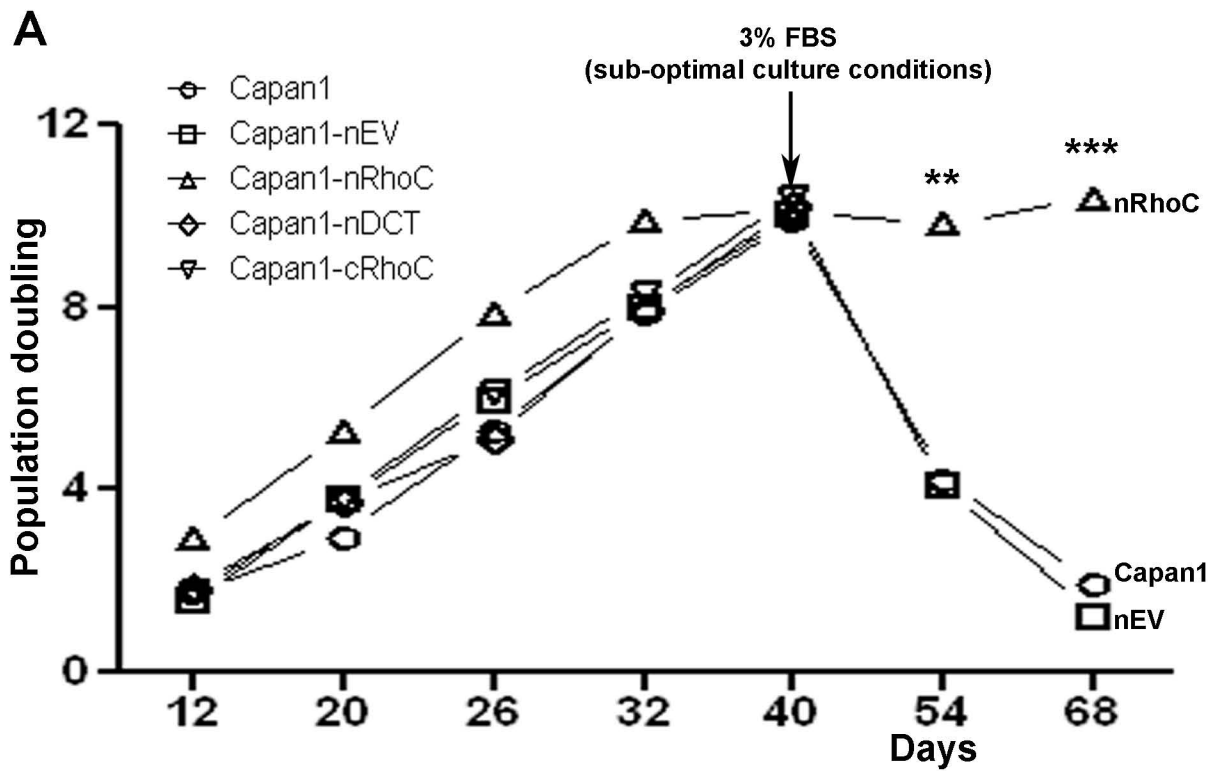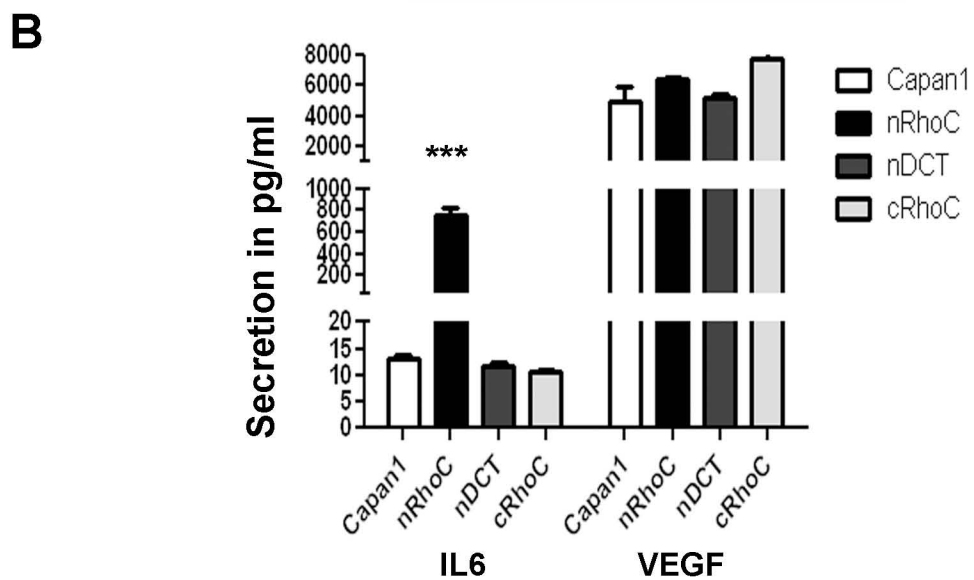

Supplement: Figure S9 — Alteration in survival after overexpression of exogenous RhoC. (A) Growth curve analysis of parental Capan1, nRhoC, cRhoC, nDCT and nEV cells showed no difference in population doublings under optimal culture conditions (10% FBS); however, under sub-optimal conditions (3% FBS, performed on day 40 onwards for Capan1, nEV and nRhoC cells) nRhoC cells sustained growth while parental Capan1 and nEV cells decreased population doublings dramatically. (**p<0.001, Student’s t-test, error bars: SE). (B) Elisa (Mesoscale) analysis of supernatant demonstrates that nRhoC secretes high amounts of IL6 as compared to parental Capan1, nDCT, cRhoC cells or nEV (not shown). Other interleukins (IL1, IL2, IL8) and growth factors (VEGF (shown), EGF, FGF) showed no such change in expression upon transfection with various distinct RhoC constructs. (*p<0.01, Student’s t-test, error bars: SE). . (PDF) [file pone.0081575.s014.pdf]

## Supplementary Figure 10

KPC mice PDAC

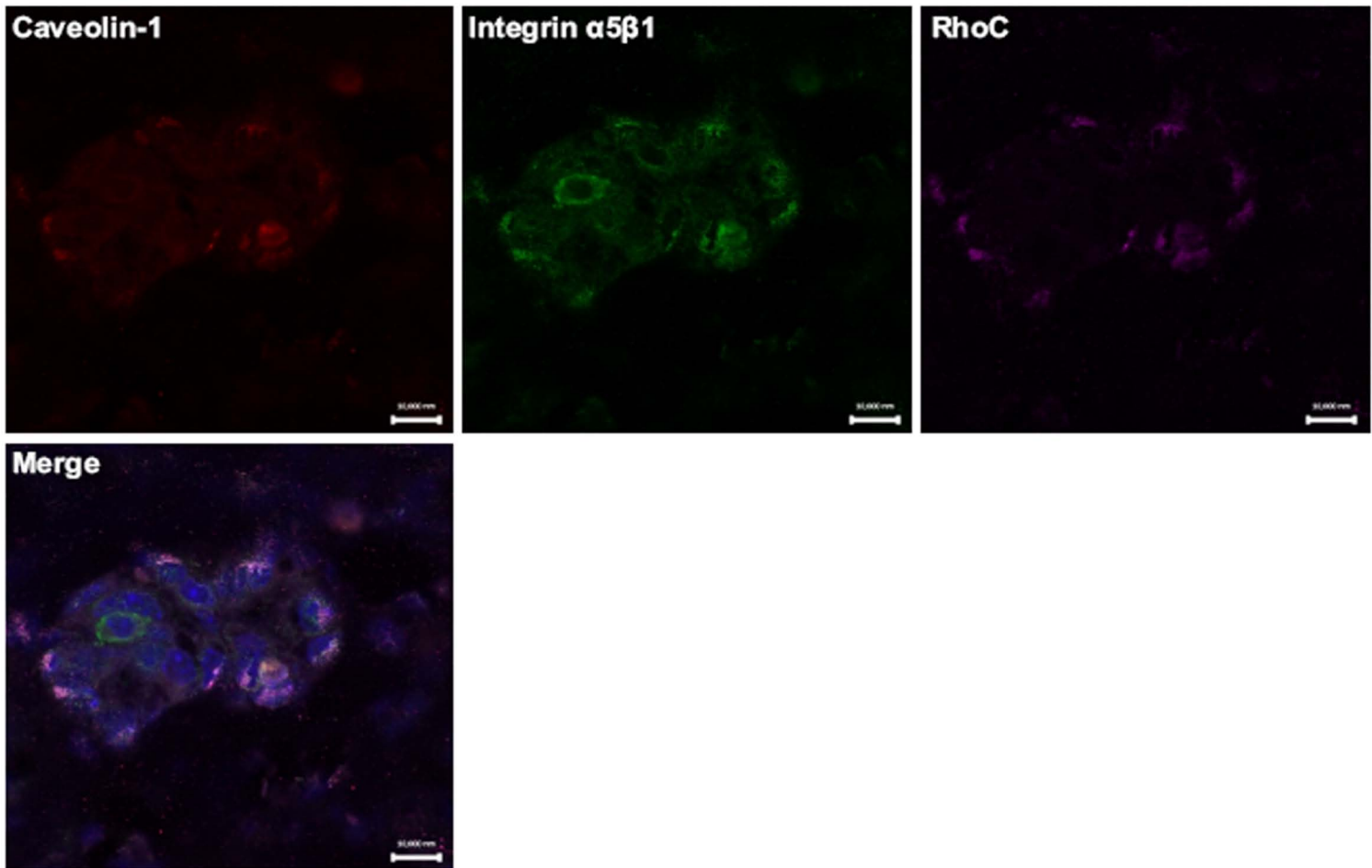

Supplement: Figure S10 — Expression pattern of Caveolin-1 integrin a5β1 and RhoC in KPC mice tumors. KPC mice tumors were stained with anti-caveolin-1, anti-integrin a5β1, and anti-RhoC antibodies. Partial co-localization of RhoC (purple) and caveolin-1 (red) can be seen in epithelial tumor cells. Co-localization of integrin a5β1 and RhoC in KPC mice tumors was almost universal. More detail in Figure S12 after quantification. Scale bar: 10 μm. (PDF) [file pone.0081575.s015.pdf]

Supplementary Figure 12

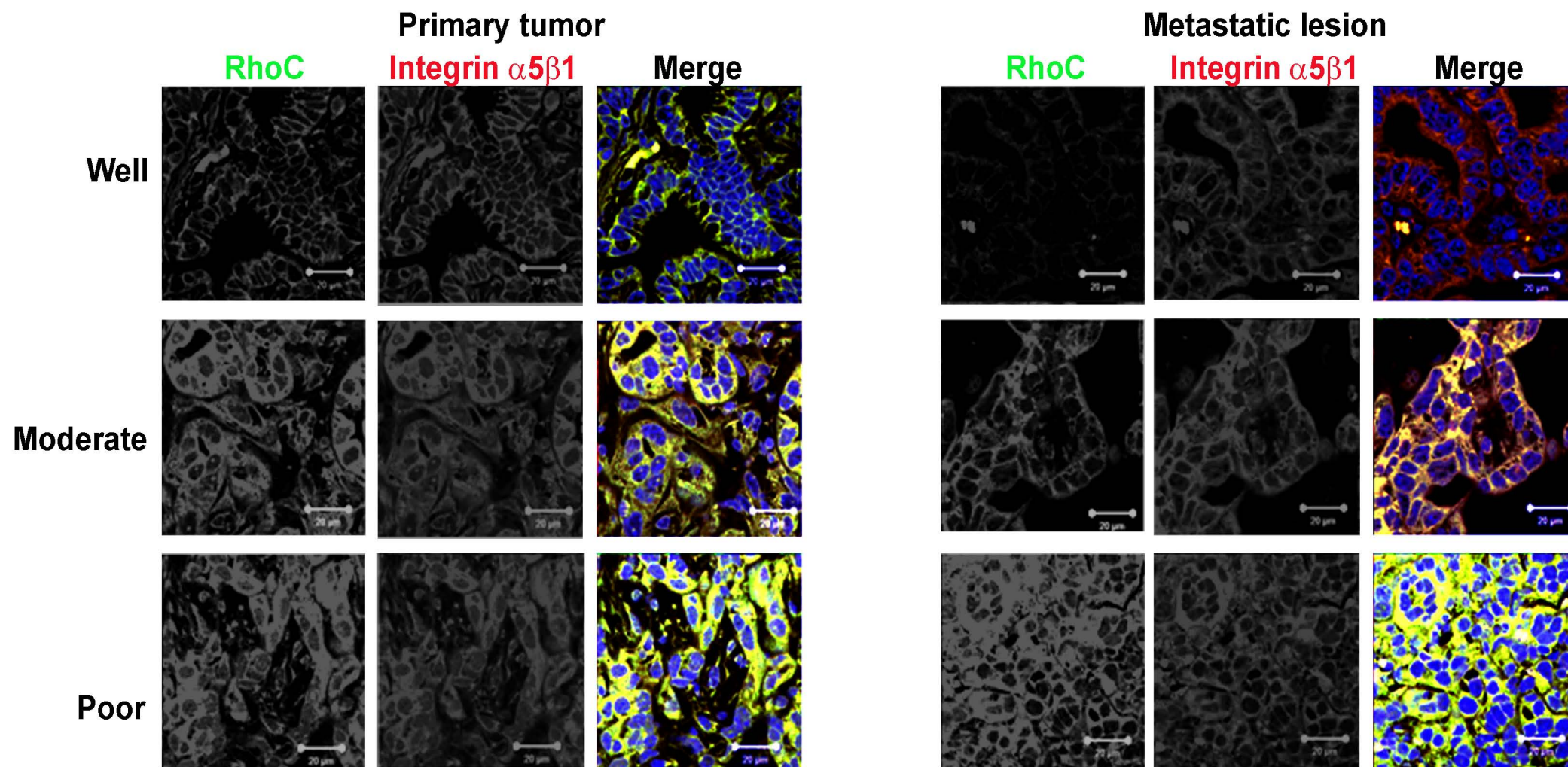

Supplement: Figure S12 — Co-localization of RhoC and integrin α5β1 correlated with poor differentiation status and enhanced metastatic potential in transgenic mouse pancreatic ductal adenocarcinoma (PDAC) lesions. Increase in co-localization of RhoC and integrin α5β1 frequently was observed in poorly-differentiated tumors from the transgenic pancreatic cancer mouse model (LSL-KrasG12D/+;LSL-Trp53R172H/+;Pdx-1-Cre transgenic mice). This observation appeared more obvious in metastatic tumors rather than in primary ones. Metastatic lesions: well- and moderately-differentiated lesions were from liver metastasis, poorly-differentiated lesion was from lung metastasis (Green: RhoC; Red: integrin α5β1; Blue: DAPI; Merge: the co-localized sites in yellow). Scale bar: 20µm. (PDF) [file pone.0081575.s017.pdf]

**Supplementary Figure 13**

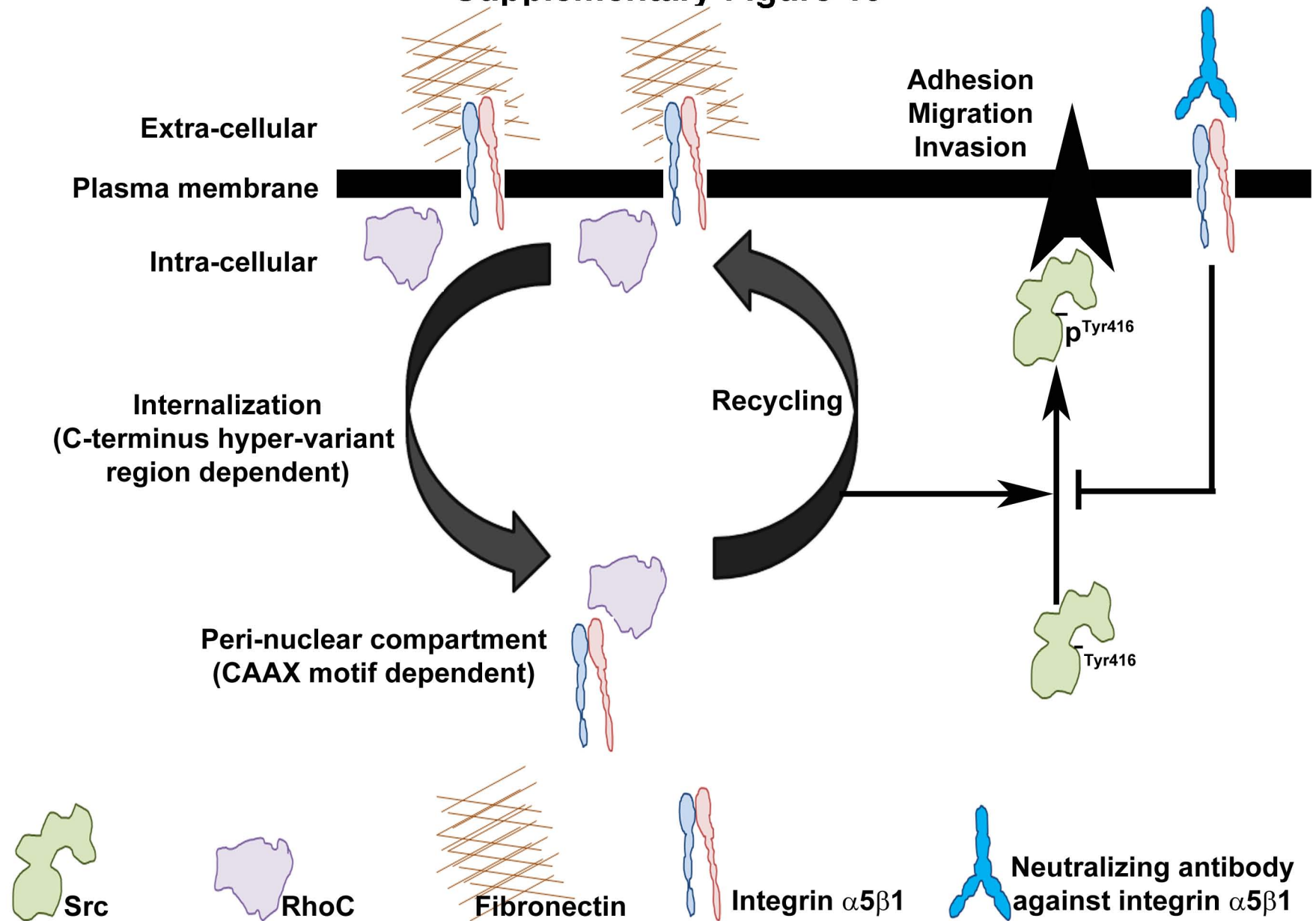

Supplement: Figure S13 — Model of suggested RhoC-integrin α5β1-Src interactions. RhoC interacts with integrin α5β1 and enhances its trafficking upon fibronectin adherence; this subsequently activates downstream Src. The interaction of RhoC and integrin α5β1 relies on the intact C-terminus divergent region of RhoC, while the translocation of the interacting RhoC- integrin α5β1 to the peri-nuclear region requires a CAAX motif-dependent, post-translational modification. Disruption of the internalization of integrin α5β1, by applying neutralizing antibody, or disruption of the recycling of integrin α5β1 by removal of CAAX motif-dependent membrane localization, abrogates the subsequent Src activation leading to a decrease in RhoC-enhanced cell migration. (PDF) [file pone.0081575.s018.pdf]
