# Supplementary material for: RhoC Interacts with Integrin α5β1 and Enhances Its Trafficking in Migrating Pancreatic Carcinoma Cells
Source: PLoS One. 2013 Dec 3;8(12):e81575. doi: 10.1371/journal.pone.0081575 (PMC3849283; doi:10.1371/journal.pone.0081575)
Supplement: Figure S11 — Co-localization analysis of RhoC and integrin α5β1 using LSM710 Zen software. The confocal image of a double-stained tissue section was loaded into Zen software in the ‘co-localization’ window. The Y-axis presents the RhoC staining intensity (green channel), and the X-axis presents the integrin α5β1 staining intensity (red channel). Only pixels that exist in both channels were plotted into the density plot. The images of normal pancreatic tissues were used to establish the base line defining the plot regions. Polygons were drawn to define the ROI (region of interest). Each ROI generated a density plot with region 1 (red channel above baseline), region 2 (the green channel above baseline) and region 3 (the overlap signals above baseline). Relevant data for each of the 1, 2 and 3 regions of each ROI were presented in the table of the analysis window. The data from region 3, the positive Co-localization region, were collected for calculating the ‘Co-localization Index’. Co-localization Index was calculated by multiplying region 3 data ‘Relative area (%)’, ‘Mean intensity ChS1-T2’ (RhoC mean intensity), ‘Weighted Coloc.Coefficient ChS1-T2’ (RhoC co-localization coefficient) and ‘Overlap Coefficient’ (RhoC and integrin α5β1 overlap coefficient). The index of normal tissue was zero since the baseline was set to ensure their ‘Relative area (%) of region 3’ was at zero level, and all malignant lesions were analyzed using the same baseline setting. Red squares highlighted the data used for the calculation of ‘Co-localization Index’. (PDF) [file pone.0081575.s016.pdf]

Supplementary Figure 11

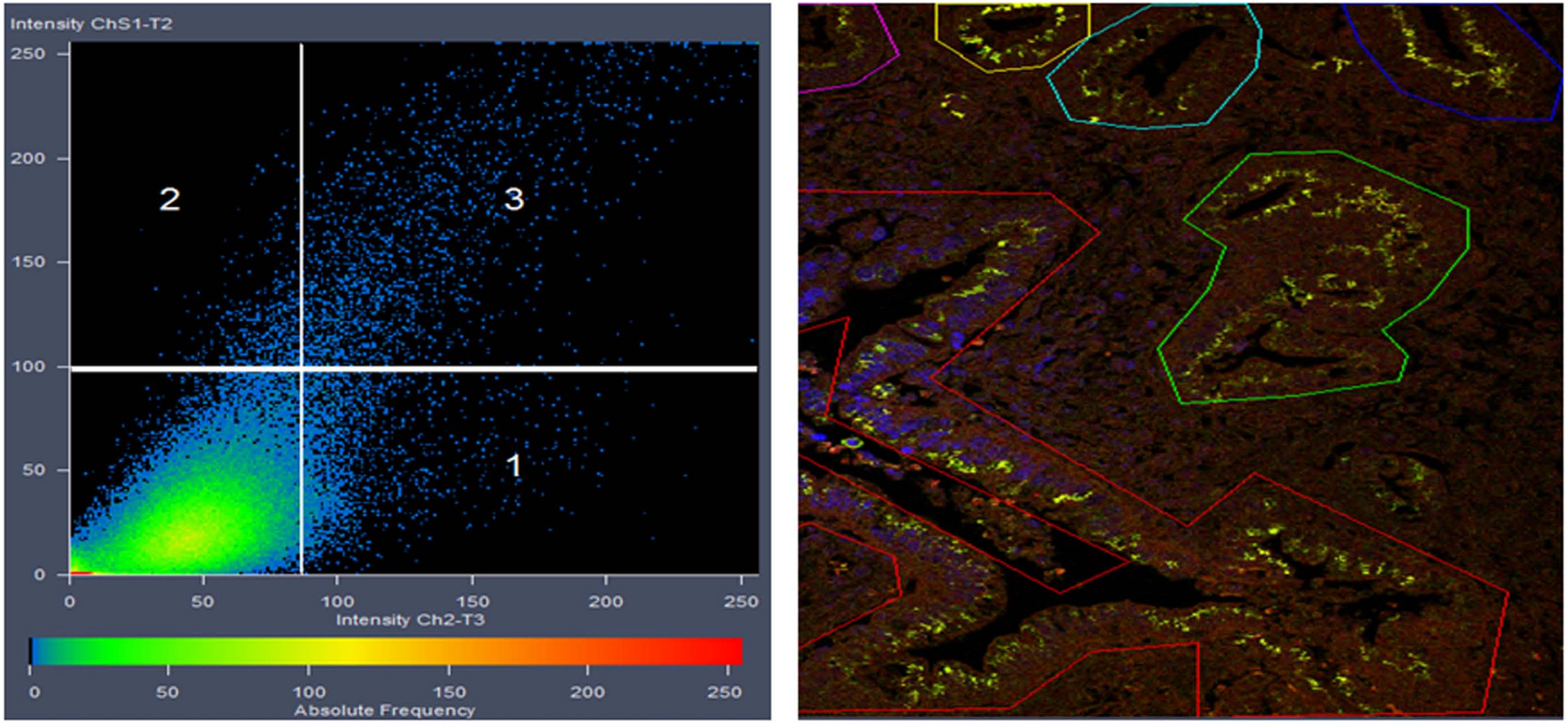

Scatter Region	Image Region	Number Pixels	Area [μm x μm]	Relative Area [%]	Mean Intensit Ch2-T3	Mean Intensity ChS1-T2	Standard Deviation Ch2-T3	Standard Deviation ChS1-T2	Colocalization Coefficient Ch2-T3	Colocalization Coefficient ChS1-T2	Weighted Coloc. Coefficient Ch2-T3	Weighted Coloc. Coefficient ChS1-T2	Overlap Coefficient	Correlation R	Correlation R x R
1	1	2153	4122.64	1.3	102	49	21	24							
2	1	344	658.70	0.2	73	120	10	21							
3	1	1173	2246.10	0.7	125	157	33	42	0.353	0.773	0.402	0.817	0.86	0.63	0.40
1	2	235	449.99	1.0	97	64	12	24							
2	2	88	168.51	0.4	72	117	12	16							
3	2	315	603.17	1.3	124	149	29	37	0.573	0.782	0.632	0.820	0.87	0.66	0.43
1	3	80	153.19	1.0	99	71	11	20							
2	3	23	44.04	0.3	73	123	9	17							
3	3	139	266.16	1.7	134	151	38	39	0.635	0.858	0.701	0.881	0.90	0.74	0.55
1	4	38	72.76	1.1	98	72	11	23							
2	4	26	49.79	0.8	70	126	13	21							
3	4	149	285.31	4.4	149	181	44	43	0.797	0.851	0.856	0.892	0.91	0.69	0.47
1	5	36	68.93	0.4	100	75	11	18							
2	5	13	24.89	0.2	70	112	12	11							
3	5	54	103.40	0.6	126	158	35	39	0.600	0.806	0.654	0.854	0.90	0.76	0.58
1	6	30	57.45	0.5	96	69	11	23							
2	6	22	42.13	0.4	66	120	12	20							
3	6	23	44.04	0.4	112	156	23	35	0.434	0.511	0.472	0.575	0.85	0.58	0.34
